# An efficient post-hoc integration method improving peak alignment of metabolomics data from GCxGC/TOF-MS

**DOI:** 10.1186/1471-2105-14-123

**Published:** 2013-04-10

**Authors:** Jaesik Jeong, Xiang Zhang, Xue Shi, Seongho Kim, Changyu Shen

**Affiliations:** 1 Department of Biostatistics, 410 West 10th st., Indianapolis, IN 46202, USA; 2 Department of Chemistry, 2320 South Brook Street, Louisville, KY 40292, USA; 3 Department of Bioinformatics and Biostatistics, 485 E. Gray St, Louisville, KY 40292, USA

## Abstract

**Background:**

Since peak alignment in metabolomics has a huge effect on the subsequent statistical analysis, it is considered a key preprocessing step and many peak alignment methods have been developed. However, existing peak alignment methods do not produce satisfactory results. Indeed, the lack of accuracy results from the fact that peak alignment is done separately from another preprocessing step such as identification. Therefore, a post-hoc approach, which integrates both identification and alignment results, is in urgent need for the purpose of increasing the accuracy of peak alignment.

**Results:**

The proposed post-hoc method was validated with three datasets such as a mixture of compound standards, metabolite extract from mouse liver, and metabolite extract from wheat. Compared to the existing methods, the proposed approach improved peak alignment in terms of various performance measures. Also, post-hoc approach was verified to improve peak alignment by manual inspection.

**Conclusions:**

The proposed approach, which combines the information of metabolite identification and alignment, clearly improves the accuracy of peak alignment in terms of several performance measures. R package and examples using a dataset are available at http://mrr.sourceforge.net/download.html.

## Background

High-throughput technology generates a large volume of high dimensional data that require efficient and accurate bioinformatics tools to extract useful information. The comprehensive two dimensional gas chromatography mass spectrometry (GCxGC/TOF-MS), a powerful high-throughput technology for metabolomics, produces data with much improved separation capacity, signal-to-noise (SNR) ratio, chemical selectivity, and sensitivity [1-3]. Yet, data preprocessing is still one of the most important factors affecting subsequent statistical analysis results [4]. Although all preprocessing steps are important, metabolite identification and peak alignment, especially in GCxGC/TOF-MS based metabolomics, have been considered key data preprocessing steps before downstream bioinformatic analysis, and have gained a lot of attention over the past two decades.

It is very common that multiple samples are analyzed for the purpose of increasing statistical confidence. In such experiments, it is crucial to recognize the peaks generated by the same compound from different samples. For this, many alignment methods for GCxGC data have been developed. They can be classified into two categories: alignment by profile and alignment by peak. Profile alignment uses raw instrument data to adjust retention times (RT) while peak alignment uses peak lists that are produced by ChromaTOF software after deconvolution of the raw instrument data. To our knowledge, four profile alignment methods have been developed so far [5-8]. The algorithms introduced in the first two papers align only local region of interest while the latter two align entire chromatogram in the two dimensional GC. However, those profile alignment methods use only the two dimensional retention times for alignment even though the fingerprint information of metabolite (i.e., mass spectrum) is readily present in the data, causing increased false alignment [1,9,10]. To remedy such a problem, several peak alignment methods, which utilize both closeness in two dimensional retention times and similarity in mass spectra, have been developed: MSort [11], DISCO [1], SW [12], mSPA [9], Empirical Bayes method [10].

The accuracy of peak alignment was increased through the development of peak alignment methods using both RT and mass spectrum information. However, those methods still have a limitation that they consider peak alignment and metabolite identification as two separate and distinct data processing steps. Such an isolated data analysis strategy makes it less efficient to remedy potential errors in each step. For instance, since experimental data are contaminated with uncontrollable noise, there is some chance that true positive pairs (i.e., pairs of peaks from two samples that are generated by the same compound) may not be aligned by peak alignment method. Indeed, peak alignment method cannot align true positive pairs if they are not the best hit during peak matching. Therefore, it is important to borrow some information from identification results to find some true positive pairs from the set of false negative pairs that are mistakenly classified by alignment. We call this process post-hoc approach.

The post-hoc approach combines two sets of aligned peak lists, i.e., one from an existing alignment method and the other from a naive peak alignment. The latter uses the name only identified by ChromaTOF software, which is a well-known sample software package with capability of performing metabolite identification from experimental data acquired on a GCxGC/TOF-MS instrument. On the other hand, among 5 peak alignment methods available, we here consider the most recent three methods: SW, mSPA and EBM. The reason is that MSort and DISCO were developed by the same group and had many properties in common, and that their nice properties were incorporated into other three methods. Here is brief introduction of how the post-hoc approach works: given two alignment results, we get a Venn diagram presenting the relationship between two results and then peak pairs in each section of the Venn diagram are further validated by applying cutoff value, which is interpreted as a confidence of similarity. By this process, some true positive pairs with high similarity that were not the best hit during peak matching can be saved, resulting in better performance.

We validate the proposed post-hoc on a mixture of standard compounds and two sets of real data from animal (mice) and plant (wheat), and also perform comparison studies in three different ways: (1) comparison before/after post-hoc analysis within each method (within-comparison); (2) comparison among three peak alignment methods (across-comparison); (3) comparing three methods to reference method (reference-comparison). Note that three existing methods such as SW, mSPA and EBM are referred to as its own name. On the other hand, the name of their post-hoc versions is followed by “post-hoc” (i.e., SW post-hoc, mSPA post-hoc and EBM post-hoc). Therefore, we consider a total of 7 peak alignment methods: 1 Naive, 3 peak alignment methods and their post-hoc versions.

We further validate our post-hoc approach by manual inspection to verify that the proposed method produces better alignment results. In addition, as a real life application of the post-hoc approach, we consider biomarker metabolite discovery. For clarity, real life application means that the data were collected from a number of biological samples with the purpose of studying a real-life biological problem. The rest of the article consists of as follows. In Results and discussion Section, we provide post-hoc results and then some conclusions are provided in Conclusion Section. In Methods Section, we summarize three existing methods. We explain our post-hoc algorithm in Algorithm Section. Finally, we summarize three datasets and explain peak merging in Experiment Section.

## Results and discussion

Before we look at results, we clarify all factors considered here: two types of peak merging (area- and similarity-based peak merging), two different cutoffs (cutoff 1 and cutoff 2) and two different performance measures (distance- and variation-based measure). Two peak merging methods use different rules. That is, area-based peak merging selects a compound with the biggest peak area as a representative peak and similarity-based peak merging is exactly the same except for using similarity instead of peak area. Cutoff 1 is applied to similarity score and cutoff 2 is applied to the number of compounds with the same name. Two performance measures, distance- and variation-based measures, base their definitions on Euclidian distance and coefficient of variation, respectively.

Regardless of peak merging methods, we see similar results and here present results for area-based peak merging only. Other results are provided in Additional files 1, 2 and 3. Additional file 1 includes experiment details, detailed description of three peak alignment methods and result plots. Additional file 2 includes all result tables about retention time-based performance measure while Additional file 3 includes all result tables about the number of aligned peaks for all combinations of two cutoffs. We consider 10 cutoff 1 values (0.5, 0.6, 0.7, 0.8, 0.85, 0.9, 0.93, 0.95, 0.97, 0.99) and different sets of cutoff 2 depending on the number of replicates of each dataset. Graphical representation of how to apply cutoff 1 and cutoff 2 is provided in Additional file 1: Figure S2.

### Performance measures

We consider two kinds of retention time-based measures: distance-based average and variation-based coefficient of variation (CV) average (i.e., the smaller the measure is, the better performance). Furthermore, we consider four different submeasures using RT marginally or jointly within each retention time-based measure.

#### Pairwise post-hoc measure

All pairwise post-hoc measures are summarized as:

(1) Distance-based average

Mean of the distance between first RTs: DRT1

Mean of the distance between second RTs: DRT2 Mean of DRT1 and DRT2: DMRT

Mean of the distance between two RTs: DRT

(2) Variation-based CV average

Mean of the CVs between first RTs: CRT1

Mean of the CVs between second RTs: CRT2 Mean of CRT1 and CRT2: CMRT

Mean of the means between the first and second CVs: CRT

#### Global post-hoc measure

All pairwise post-hoc measures are summarized as:

(1) Distance-based average

Mean of the means of the distances among first RTs: DRT1

Mean of the means of the distances among second RTs: DRT2

Mean of DRT1 and DRT2: DMRT

Mean of the means of distances among two RTs: DRT

(2) Variation-based CV average

Mean of the CVs among first RTs: CRT1

Mean of the CVs among second RTs: CRT2 

Mean of CRT1 and CRT2: CMRT

Mean of the means between the first and second CVs: CRT

### Pairwise post-hoc results

We calculate distance- and variation-based measures for all possible pairs. Since dataset1 (dataset2/dataset3) includes 10 (5/8) technical replicates, we get 45 (10/28) pairs to align. The average values of performance measures from all pairs (before and after post-hoc) are summarized in Tables 1 (distance-based measure) and 2 (variation-based measure) when cutoff 1=0.99 (DRT and CRT only). All other result tables are provided in Additional file 2.

**Table 1:** Pairwise: average of distance-based measures (DRT only) over all pairs: before/after post-hoc analysis when cutoff 1=0.99

**Method**	**Std. mixture**	**Mice**	**Wheat**
EBM	16.3451/2.4902	81.7301/13.4839	158.1628/25.4150
mSPA	5.9654/6.0653	15.8091/5.6013	25.9831/6.6434
SW	1.4624/1.7551	0.8483/7.9597	1.9651/10.400
Naive	7.3613	73.4156	160.0547

**Table 2:** Pairwise: average of variation-based measures (CRT only) over all pairs: before/after post-hoc analysis when cutoff 1=0.99

**Method**	**Std. mixture**	**Mice**	**Wheat**
EBM	0.0082/0.0037	0.0358/0.0098	0.0490/0.0094
mSPA	0.0048/0.0050	0.0122/0.0066	0.0121/0.0038
SW	0.0028/0.0027	0.0169/0.0067	0.0123/0.0049
Naive	0.0066	0.0360	0.0480

**Figure 1:**
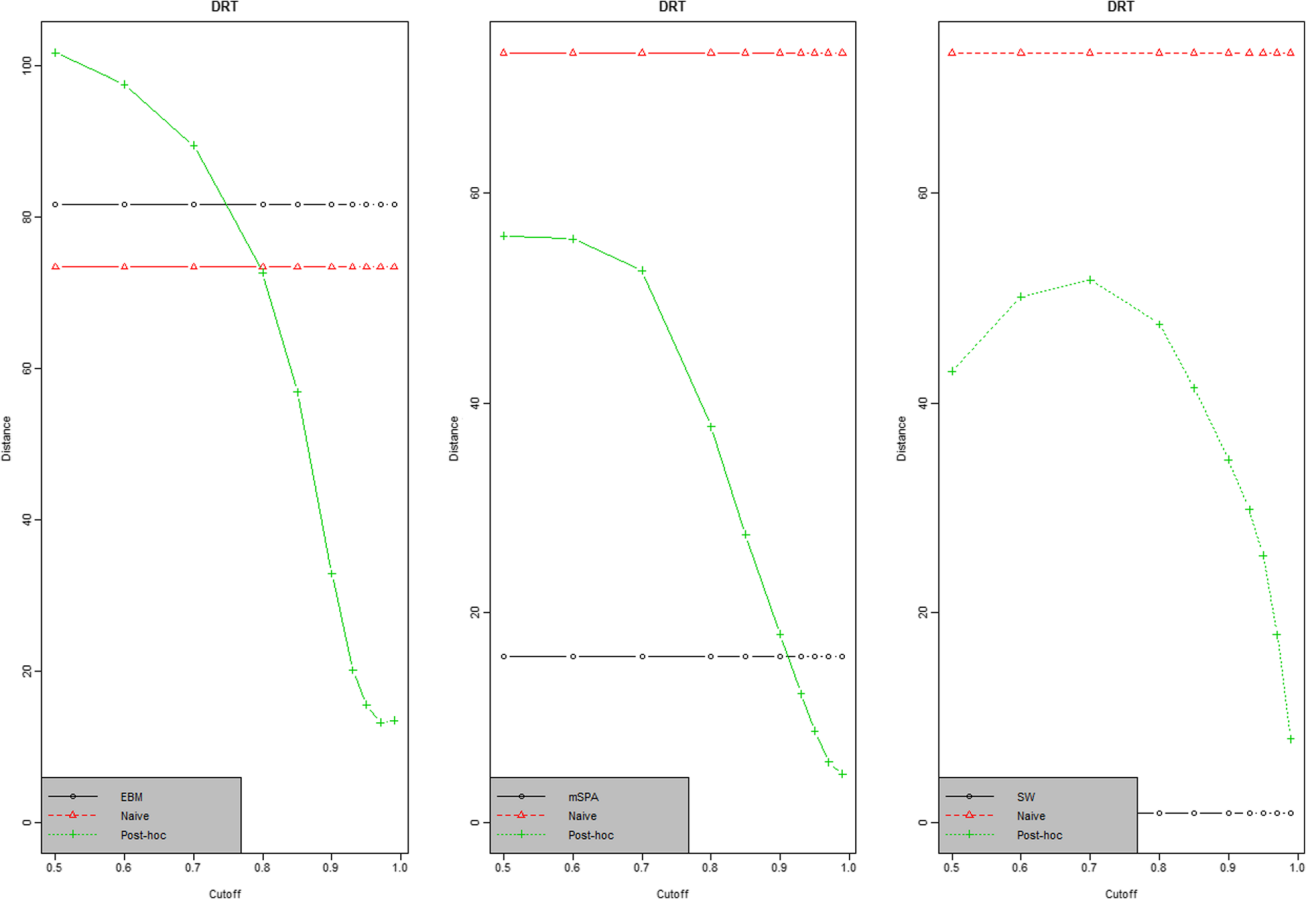
**Distance-based performance measure.** Mice data (pairwise): distance-based performance measure for EBM (the first column), mSPA (the second column) and SW (the last column), respectively. In each plot, there are 3 lines in different colors: the line in red is reference line obtained by Naive method, and black and green lines correspond to before/after post-hoc from each method.

Given a cutoff 1 value, performance measure for each experiment pair is calculated and then average over all pairs is calculated. To see the effect of cutoff 1 on performance measure, we provide trace plots of performance measure over cutoff 1. Figure 1 presents how the mean performance measures vary over cutoff 1 values. Plots for distance-based measure (mice data only) are given in the figure and other plots are provided in Additional file 1. A trend in post-hoc results is observed. As expected, EBM and mSPA post-hoc results showed the monotone decreasing trend as cutoff 1 increases because those methods employ scoring system using both retention time and mass spectra information. However, the SW post-hoc results show different pattern, i.e., quadratic structure for real biological data. The reason is that SW uses spectra-based scoring system while performance is evaluated by retention time-based measure. That is, increasing cutoff 1 that is applied to spectrum-based score does not monotonically increase the performance of retention time-based measure. However, after some high level of cutoff 1 value (say, 0.8), SW post-hoc also shows similar monotone decreasing trend.

Regarding within-method comparison, we see lots of improvement for all methods except SW on all datasets. The reason SW post-hoc has the worse result is that SW uses the retention time information more strictly, i.e., it works under the constraints that the elution order of each peak is preserved across all the experiments. However, after post-hoc, some peaks were added from the Naive method, which ignored the preservation of the elution order. This process compromises the performance measure as shown in Table 1. Regarding reference-comparison, all methods perform much better than the Naive method (performance by Naive method is represented in red in Figure 1). For comparison among three methods, we see similar performance at high level of cutoff 1 even though there is some difference at low level of cutoff 1. From another angle, we calculated the number of peaks aligned by each method. More specifically, we calculated the median value of the number of aligned peaks obtained from all possible experiment pairs. Here we provide a table summarizing results for area-based peak merging when cutoff 1=0.99 (Table 3). Complete results are provided in Additional file 3.

**Table 3:** The number of aligned peaks before/after post-hoc analysis based on area-based peak merging when cutoff 1=0.99

	**Pairwise**			**Global**		
	**EBM**	**mSPA**	**SW**	**EBM**	**mSPA**	**SW**
Std	70/61	72/71	70/67	47/44	65/63	53/63
Mice	287/142	314/166	297/155	116/65	178/87	33/65
Wheat	377/170	339/232	281/169	99/68	161/84	30/61

The number after pairwise post-hoc is the median value for all experiment pairs. For global alignment, cutoff 2 where all aligned compound have the same name is used, i.e., cutoff 2=10 (std.), =5 (mice) and =8 (wheat), respectively. The first number (before post-hoc) is the number of aligned peaks obtained by each method alone.

**Figure 2:**
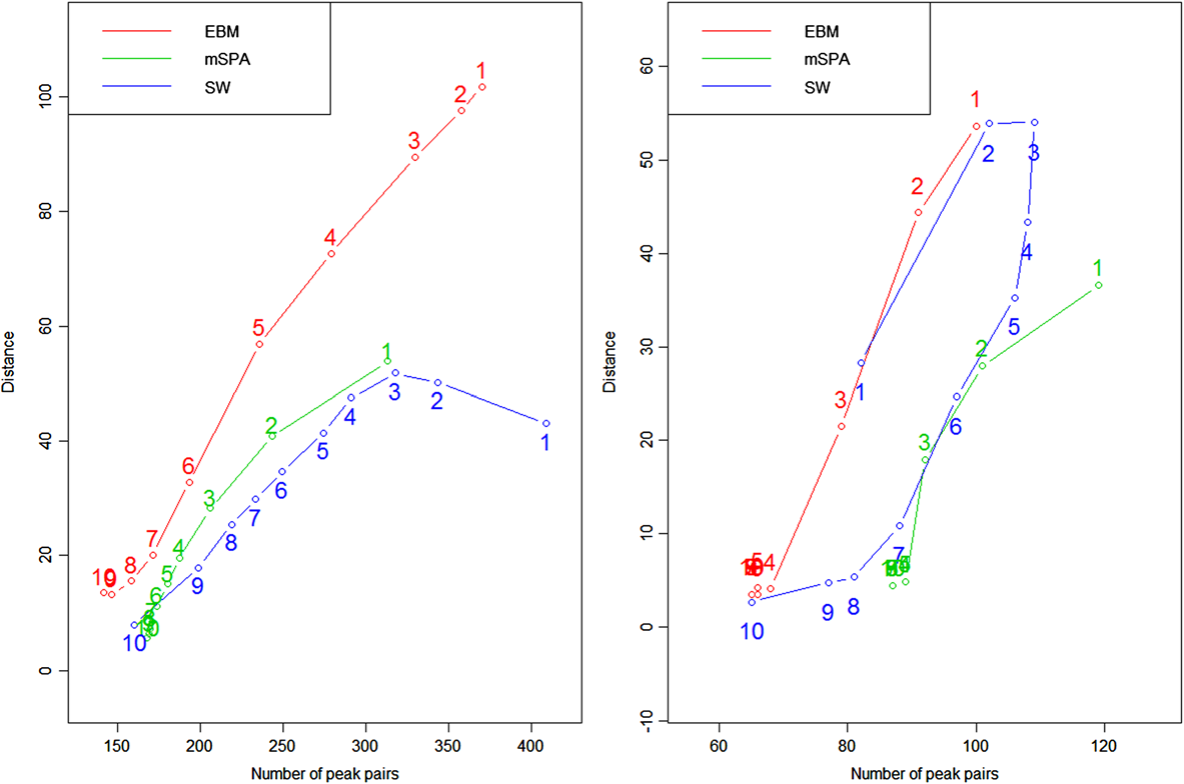
**Plot of performance measure v.s. the number of peaks.** Mice data: plot of performance measure v.s. the number of peaks, left (pairwise) and right (global). Each circle corresponds to each cutoff 1 value. Numbers from 1 to 10 correspond to each cutoff 1 (cutoff 1=0.5 (1) to cutoff 1=0.99 (10)). Roughly speaking, each curve moves from top right corner to bottom left.

Considering two sets of results together (distance-based measure and the number of aligned peaks), we noticed that (1) the number of aligned peaks decreases as cutoff 1 increases (2) since peak pairs with high similarity have more chance to survive a cutoff 1, fewer peaks with high similarity remain and performance is getting better as the number of aligned peaks decreases. Figure 2 (left panel) illustrates such relationship between distance-based performance and the number of aligned peaks as cutoff 1 increases. As seen in the figure, even though the trace plots for each method start different position (top right of the figure when cutoff 1=0.5), their ending positions (cutoff 1=0.99) are very close. That is, we see similar performance after post-hoc when high cutoff 1 value is applied.

### Global post-hoc results

For global post-hoc analysis, we consider each dataset as a series of data observed at different time points and calculate performance measures for the data: length of 10 (5/8) for dataset1 (dataset2/dataset3), respectively. For global alignment, we introduce another cutoff called cutoff 2, which plays as a tuning parameter. Cutoff 2 is applied to each of globally aligned peaks to allow some tolerance when making decision of global alignment status, i.e., correct/incorrect alignment. To see the effect of cutoff 2 on performance, different sets of cutoff 2 for each dataset were considered, i.e., 10,… , 6 for dataset1, 5,… , 3 for dataset 2, and 8,… , 5 for dataset3.

**Figure 3:**
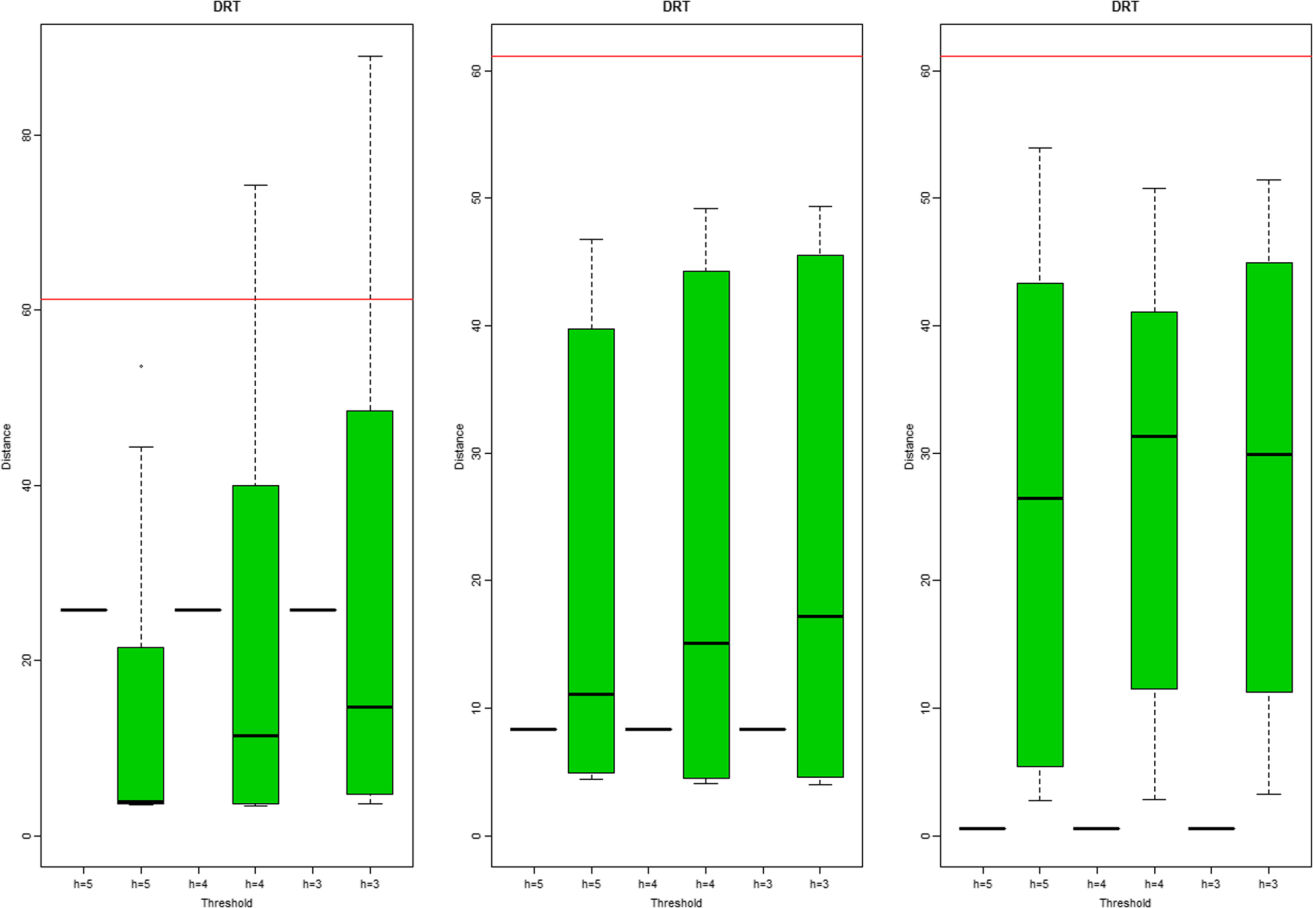
**Distance-based performance measure.** Mice data (global): distance-based performance measure for EBM (the first column), mSPA (the second column) and SW (the last column), respectively. The solid line in red presents the Naive method. Given a cutoff 2, we have two results before/after post-hoc and two box plots correspond to a cutoff 2. Box plot after post-hoc is made by using 10 numerical values corresponding to each cutoff 1 value while corresponding plots before post-hoc look like line because the results are not affected by cutoff 1.

For each cutoff 2, 10 global performance measures, each corresponding to 10 cutoff 1 values, are calculated and then a box plot is made using those 10 performance measures. However, since performance measure before post-hoc is not affected by cutoff 1, just one numerical value is available for each cutoff 2 and corresponding box plot looks like a line. In Figure 3, there are two box plots for each cutoff 2, i.e., before and after post-hoc. We also added a reference line in red, which is a performance measure obtained by the Naive method. Here we provide box plots for distance-based measure on mice data only, and boxplots for other cases are provided in Additional file 1.

Regarding reference-comparison for standard mixture data, the performance difference between each peak alignment method and the Naive method is not big (see Additional file 2). However, as the complexity of the data increases, the difference is getting more apparent. For comparison among three methods, we consider two different values: median and mean. In terms of median, for standard mixture data, EBM post-hoc shows the best results when cutoff 2=10 while mSPA or SW post-hoc provides better performance for other cutoff 2 values. For complicated data, EBM post-hoc is the best for all cutoff 2 values even though the difference among methods is not substantial (Figure 3). However, in terms of mean, we see little bit different results. The mSPA post-hoc is the best for standard mixture and SW post-hoc for real biological data (right panel of Figure 2).

From a different perspective, we calculated the number of peaks aligned by each method. Cutoff 2 where all aligned compounds have the same name was selected, i.e., cutoff 2=(10,5,8) for each dataset, respectively. Here we provide a table summarizing results for area-based peak merging when cutoff 1=0.99 (Table 3). More results are provided in Additional file 3.

As mentioned in pairwise post-hoc results Section, the reason SW has worse results after post-hoc is that SW has rigorous control on the alignment quality in terms of retention time. Therefore, it is possible for the post-hoc analysis to compromise the retention time performance as the Naive method does not use retention time information. In addition, because of the rigorous control, SW post-hoc tends to have less aligned peaks as compared with other methods (particularly for global alignment), but more aligned peaks when compared with SW itself. This kind of trade-off between SW and SW post-hoc can be interpreted as the sensitivity versus specificity issue.

Similar to pairwise post-hoc results, we noticed that the number of aligned peaks decreases as cutoff 1 increases. As expected, the performance is getting better as the number of aligned peaks decreases. Figure 2 (right panel) illustrates the relationship between distance-based measure and the number of aligned peaks for each cutoff 1 when cutoff 2=5. Not surprisingly, all alignment methods converge to the left bottom of the figure, implying that less peak pairs with high quality remain. Combining all results together, mSPA post-hoc performs slightly better than the other two even though the difference in performance is not substantial.

### Manual validation of peak alignment by post-hoc

To investigate the performance of peak alignment by post-hoc, we manually inspected some aligned peaks by using raw chromatogram data including 3D plot. For this, we selected a pair of experiments from standard mixture data. We then applied EBM method to the experiment pair and got 67 aligned metabolites. Similarly, we got 59 metabolites by EBM post-hoc, i.e., 8 of 67 were removed. Among those 8, 6 were verified to be incorrectly aligned (i.e., removal by post-hoc was correct decision) and the other 2 correctly aligned. To provide some evidence supporting such validation, we selected 2 of 8 removed pairs (i.e., one of them is correctly removed and the other one is incorrectly removed by post-hoc) and provided corresponding 3D chromatogram plots in Additional file 1: Figure S5. As a result, manual inspection supported that our post-hoc approach improved peak alignment.

### Application to metabolite biomarker discovery

As a real life application of our post-hoc approach, we consider biomarker metabolite findings. For this purpose, we analyzed a new dataset consisted of two groups: 6 low fat diet mice (LFD) and 5 high fat diet mice (HFD). Briefly, liver tissue samples were collected from 11 mice and subjected to the GCxGC/TOF analysis to identify metabolites differentiation as the consequence of the two diets. The detailed description of the data is provided in Additional file 1. The process of biomarker discovery is summarized:

(1) apply global alignment method to the data

(2) apply multivariate statistical analysis to the aligned peaks.

More specifically, we first obtain globally aligned peaks and then find some statistically significant metabolites at given nominal level (say FDR=0.05).

With the data, we obtained 49 aligned metabolites by EBM when cutoff 2=11. After that, the significance analysis of microarray (SAM) [13] was applied to the abundance of the 49 metabolites. At the FDR=0.05, we got a total of 13 biomarker metabolites whose abundance are significantly different between two groups. For 7 of them, abundance of HFD is higher than that of LFD. The list of 13 biomarker metabolites is provided in Table 4 (left 2 columns). After applying post-hoc process to the data, we obtained 44 globally aligned metabolites when cutoff 1=0.99 and cutoff 2=11. The SAM was applied to the abundance of the 44 metabolites. At the FDR=0.05, we got a total of 15 biomarker metabolites whose abundance was significantly different between two groups. For 10 of them, abundance of HFD is higher than that of LFD. The list of 15 biomarker metabolites is provided in Table 4 (right 2 columns). In addition, we found 10 common metabolites in both biomarker sets: 6 (higher abundance in HFD) and 4 (higher abundance in LFD). Those 10 metabolites are represented by asterisk * right after the CAS name (Table 4). More results such as SAM score before/after post-hoc are provided in Additional file 1.

**Table 4:** 13/15 biomarker metabolites before/after post-hoc

**Before**		**After**	
**Name**	**CAS**	**Name**	**CAS**
Acetophenone	98-86-2*		
Palmitelaidic acid	82326-15-6*		
N,N-Diethyl-1,1,1-trimethylsilylamine	996-50-9*		
Tetradecanoic acid	104255-79-0*		
L-Phenylalanine	107715-95-7*		
Arachidonic acid	113516-18-0*		
Ethanol	2916-68-9	L-Tyrosine	107716-01-8
		L-Glutamic acid	107715-97-9
		Propanedioic acid	18457-04-0
		Butane	2568-90-3
Pyridine	110-86-1*		
Cyclotrisiloxane, hexamethyl-	541-05-9*		
Ethanamine	16654-64-1*		
Dodecanoic acid	104255-77-8*		
L-Lysine	107715-99-1	Sulfuric acid	85207-88-1
Pentasiloxane	141-63-9		

Peak alignment method EBM is applied to diet data with two group (HFD and LFD) and then SAM is applied to 49 globally aligned metabolites. By SAM, 13 biomarker metabolites are found. For 7 of them, the abundance of HFD is significantly higher than that of LFD (top 7 metabolites); Global post-hoc with cutoff 1=0.99 and cutoff 2=11 is applied to diet data with two group (HFD and LFD) and then SAM is applied to 44 globally aligned metabolites. By SAM, 15 biomarker metabolites are found. For 10 of them, the abundance of HFD is significantly higher than that of LFD (top 10 metabolites). Compared to the results before post-hoc, we got more biomarker metabolites. 10 common biomarker metabolites in both results are denoted by * right after the CAS name.

## Conclusions

Even though many peak alignment methods have been developed, they have a limitation that they consider the best hit only during peak matching, resulting in decreased accuracy. To overcome such a limitation, we introduced a novel post-hoc approach to integrate identification and peak alignment. Through the comparison before/after post-hoc analysis within each method, we noticed that the problem caused by considering the best hit only has partly been solved by post-hoc approach in that we see some improvement on the performance measures. Especially, in case of standard mixture data, we see dramatic change in performance measure for EBM. On the other hand, in case of complicated data, we see a lot of improvement in mSPA and EBM post-hoc results.

Through the comparison among three peak alignment methods, we noticed that, even though there was big difference in performance among three methods, such a big difference disappeared after post-hoc. In other words, the efficiency of any peak alignment method can be elevated by post-hoc method so that all methods have similar performance in the end, which is another good property of post-hoc.

We considered two different ways of peak merging: peak merging by area and peak merging by similarity.Two peak merging results for real data were very similar (i.e., the range of concordance is 86.3 to 88.7% for mice and 83.5 to 86.5% for wheat, respectively) and the effect of peak merging on performance was not substantial. That is, we noticed that there were similar overall patterns in the results obtained by both peak merging even though there exists slight difference in magnitude.

In the pairwise post-hoc, SW post-hoc results show different trend from the other two, implying that the effect of post-hoc approach varies according to both scoring system involved in the peak alignment method and performance measure. However, after some high cutoff 1, the effects of such factors disappear, i.e., all methods show similar trend.

Even though we considered homogeneous experiment only in the paper, the proposed idea can be applied to heterogeneous experiment as well. However, it is necessary to develop new performance measure suitable for heterogeneous experiment, which is done under different experimental conditions.

As one of reviewers suggested, we manually validated our post-hoc approach on a pair of standard mixture data and noticed that the proposed method improved peak alignment. As a real life application, we considered biomarker metabolite findings. In this example, we found 15 biomarker metabolites with statistically significant difference in abundance between two groups. Compared to the results before post-hoc, we got more biomarker metabolites after post-hoc. However, utility of selected biomarker metabolites need to be further studied and then might be used for other analysis.

## Methods

We briefly introduce three peak alignment methods that utilize the output of ChromaTOF software as input: SW, mSPA and empirical Bayes method (EBM). Detailed explanation of the methods and methodology comparison among them are provided in Additional file 1.

### Naive and three existing peak alignment methods

#### Naive method

The naive method uses the name identified by ChromaTOF software for alignment purpose. In other words, given a pair of experiments, compounds with the same name are aligned.

#### Smith-Waterman (SW)

Smith and Waterman developed a general method for identification of molecular subsequences [14]. Kim et al. [12] modified the traceback process of the SW method and proposed three variants of the algorithm.

Given two peak lists to align, the SW algorithm produces a matrix representing the degree of similarity with a boundary condition and use the matrix for peak alignment. They consider Pearson's correlation coefficient as similarity measure.

#### mSPA

The method consists of two main algorithms: peak matching and parameter optimization. As a similarity measure for peak matching, they defined a mixture similarity score, which is a mixture of mass spectral similarity and retention time closeness. As measure of closeness in retention time, they considered four different distance measures, definitions of which are provided in Additional file 1. They considered two spectral similarity measures, dot product and Pearson correlation.

For parameter optimization, they defined an ad-hoc likelihood-type function. The value maximizing the function is considered parameter estimate.

### Empirical Bayes model (EBM)

Jeong et al. (2011) developed a hierarchical statistical model for metabolite identification and peak alignment in an unified framework for GCxGC/TOF-MS data. To address the nature of the database search algorithm, they employed an empirical Bayes model with four layers of hierarchy: (1) marginal probability that each compound in reference exists in target is calculated (2) depending on the existence/absence of a compound, different conditional probability of the compound being matched to a compound in target is calculated. (3) based on the information from previous two layers, conditional probability that the match is correct is calculated. (4) based on the decision in layer 3, the scores are separated and then used to estimate two score density functions: true positive and true negative score densities.

For peak alignment, Jeong et al. (2012) used the posterior probability that peak matching is correct (layer 3), which is called matching confidence. Peak pairs with confidence measure larger than a cutoff prespecified are selected for alignment.

### Algorithm

**Figure 4:**
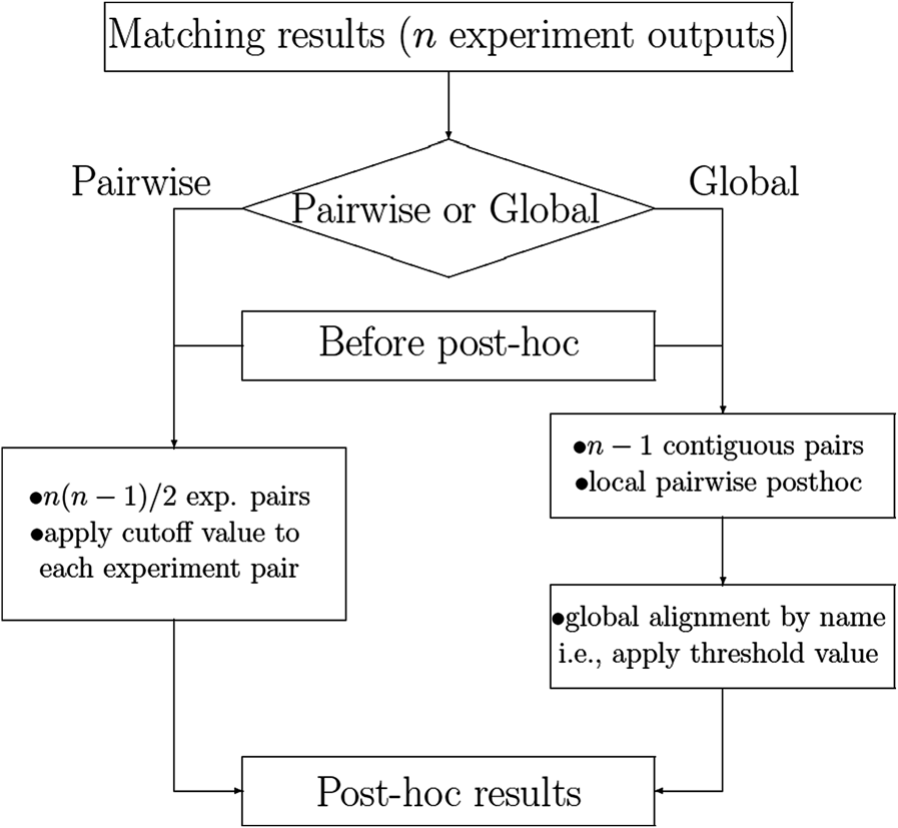
Flowchart of the process of the post-hoc algorithm.

We here consider both pairwise and global post-hoc approaches. The main difference between the two is that global post-hoc aligns more than two experimental outputs at once while pairwise post-hoc is used for a pair of outputs. That is, global post-hoc can be used for a series of data, for example, time course data observed at several consecutive time points. The flowchart of the algorithm is graphically represented in Figure 4. A typical example of the application of pairwise post-hoc is the before/after type of study to investigate the effect of a medical intervention/condition, where for each subject one biological sample is collected before the intervention/condition, and one is collected after. A typical application of global post-hoc is comparison between two groups (i.e. cases versus controls, intervention versus control), where each group is composed of multiple subjects.

### Pairwise post-hoc analysis

**Figure 5:**
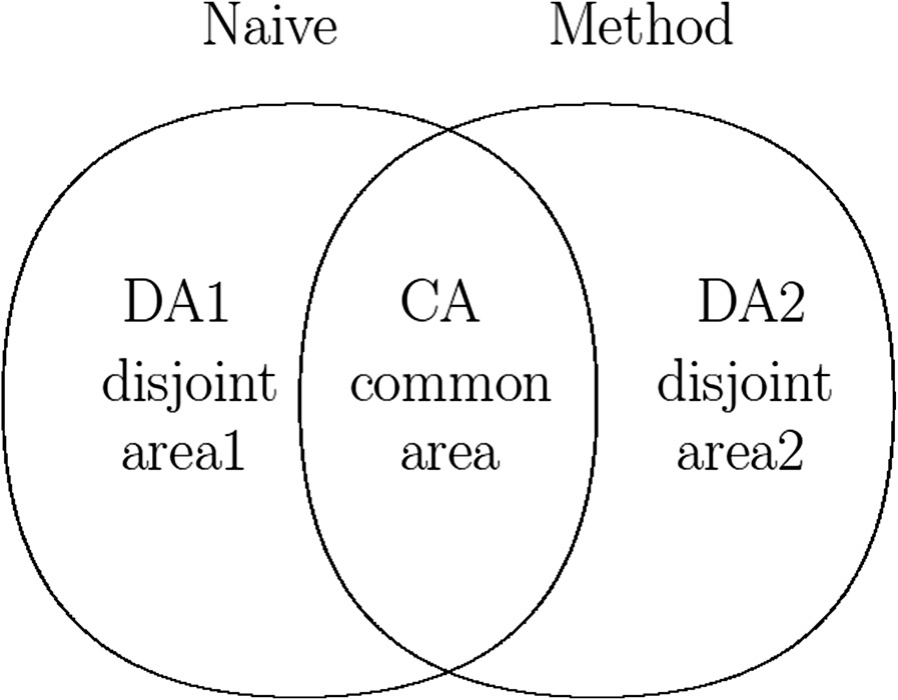
**Relationship between peak alignment results.** Venn diagram presents the relationship between peak alignment results obtained by the Naive method and a peak alignment method. Method presents a peak alignment method such as SW, mSPA or EBM.

We consider all possible experiment pairs. For instance, if we have 5 experiment replicates, then there are 10 possible experiment pairs to align. Given an experiment pair, a peak alignment method and the Naive method are applied to the pair, resulting in two different alignment results. Combining two results, we can get a Venn diagram presenting the relationship between two sets of alignment results (Figure 5): common peak pairs (CA) and disjoint peak pairs (DA1 and DA2).

The peak pairs in CA that are aligned by both methods are considered as high confident and are automatically kept in the positive set. For improvement purpose, our focus is on the other two areas: DA1 and DA2. Since metabolite pairs in DA1 have the same name assigned by ChromaTOF, we cross-check the pairs through matching score, which is obtained by using scoring system of peak alignment method. That is, we apply cutoff 1 to the matching score to decide if we keep or discard them. For this, we consider 10 cutoff 1 values (0.5, 0.6, 0.7, 0.8, 0.85, 0.9, 0.93, 0.95, 0.97, 0.99). If a peak pair has a matching score greater than cutoff 1 specified, the pair is considered as correct match and added to the positive set. Regarding DA2, we do the same thing with the same cutoff 1 and selected pairs are added to the positive set.

**Figure 6:**
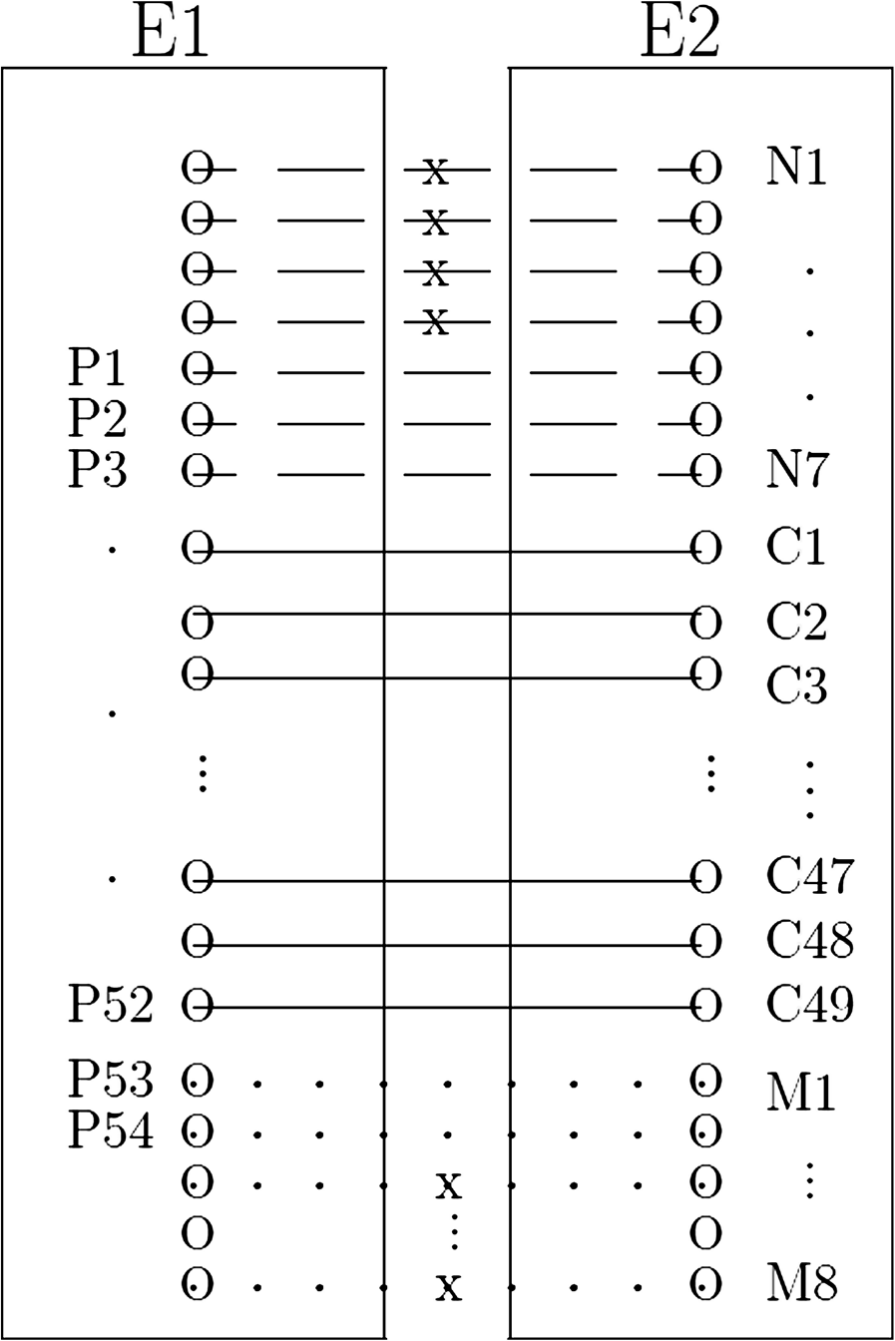
**Graphical representation of pairwise peak alignment.** Pairwise alignment: E1 and E2 are experimental outputs. 49 solid lines (denoted by C_1_,...,C_49_) presents peak matching, which were aligned by both methods. 7 dashed lines and 8 dotted lines present matched peaks by the Naive method only and a peak alignment only, respectively. That is, there are 56 peak pairs by Naive and 57 by a peak alignment method and union of two peak alignment results are 64. After applying a cutoff value, 3 pairs (N_5_; N_6_; N_7_) and 2 pairs (M_1_; M_2_) were selected as correct matching by post-hoc. Final 54 peak pairs are denoted by P_1_,...,P_54_. 'x' presents 'failure to pass the cutoff 1.'

As an illustrating example, suppose that we have 7 peak pairs in DA1 (dashed line), 49 in CA (solid line) and 8 in DA2 (dotted line). Also, assume that 3 (P_1_; P_2_; P_3_) out of 7 in DA1 and 2 (P_53_; P_54_) of 8 in DA2 pass the cutoff 1 specified. Then we end up with the positive set including 54 peak pairs, which are denoted by P_1_,...,P_54_. A graphical representation is provided in Figure 6. Further details of the algorithm are provided in Additional file 1.

### Global post-hoc analysis

**Figure 7:**
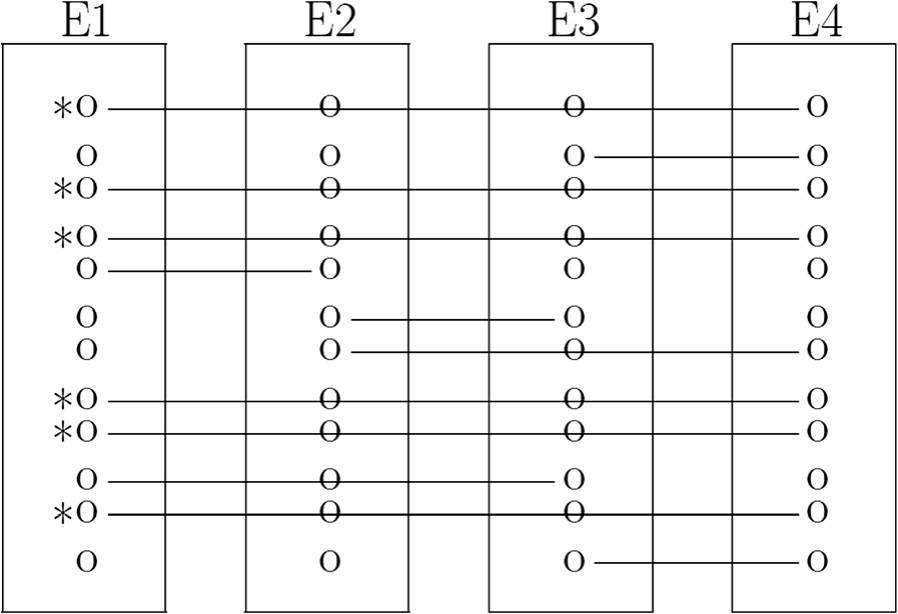
**Graphical representation of global peak alignment.** Global alignment: E1, E2, E3 and E4 are experimental outputs to align. We have 6 aligned compounds, which are denoted by *. Solid line presents peak matching.

In case of data with explicit order, we need to align all (more than two) experimental outputs in that order simultaneously. For instance, if we have 4 experimental outputs observed at different time points, then we have a series of data consisting of 3 contiguous pairs internally, i.e., (E1, E2), (E2, E3) and (E3, E4). We apply the pairwise post-hoc approach to each pair. Combining three pairwise post-hoc results, we get global matching results (Figure 7). We then select compounds with connection line through all experiments and cross-check the aligned compounds by name. Thus, there are two steps for global post-hoc algorithm (see Figure 4 for graphical representation):

Step1: apply pairwise post-hoc to each contiguous experiment pair until all pairs are done (score-based). Step2: apply cutoff 2 to all globally aligned compounds and select correctly aligned ones (name-based). To see the effect of cutoff 2 on performance, we consider different sets of cutoff 2 values for each dataset depending on the number of experiment outputs (i.e., > n/2). As an illustrating example, suppose that we have 4 experimental outputs and 6 aligned compounds (denoted by in Figure 7). Then each aligned compound has 4 names sequentially, but those names might be different. For instance, assume that an aligned compound has 3 same names, i.e., just one is different. In this case, there is some chance that the one with different name had been incorrectly identified by ChromaTOF. If it is the case, we may be able to correct the identification by replacing the possibly wrong name with the name a majority of compounds share.

### Experiments

We have three different experiment datasets: a mixture of standard compounds and two real datasets collected on mice and wheat. Experimental details are provided in Additional file 1. In case of multiple peaks, we consider two different ways of peak merging. All the mice were treated according to the experimental procedures approved by the University of Louisville Animal Care and Use Committee.

### Peak merging

Theoretically, a peak is generated by a compound. However, often we see multiple peaks in close proximity. Peak merging is used to remedy such a problem. Since peak merging by area and similarity root in different principles, two peak merging methods may produce different results. As an illustration, we selected a compound with 5 peaks called Pyridine (CAS: 110-86-1) from a mixture of standard compounds. More information for the five peaks are summarized in Table 5. As a representative peak, we select the first one if we use area-based peak merging while we get the second one if we use similarity-based peak merging.

**Table 5:** Peak selection based on area or similarity

**Name**	**CAS**	**RT1**	**RT2**	**Area**	**Similarity**
Pyridine	110-86-1	369.719	1.162	**28831918**	943
Pyridine	110-86-1	379.711	1.175	2788666	**948**
Pyridine	110-86-1	384.707	1.188	925142	931
Pyridine	110-86-1	389.704	1.208	548115	914
Pyridine	110-86-1	394.7	1.214	569849	882

Standard mixture data; peak merging by Area or Similarity. If we use area-based peak merging, the first one is selected as a representative peak.

### Peak merging results

We have 10 homogeneous experimental outputs from a mixture of 76 standard compounds, which is called dataset1. Here homogeneous means data are generated from the same biological sample under the same experimental conditions. Also, we have two sets of real data: 5 homogeneous experimental outputs from plasma of a mice (dataset2) and 8 homogeneous data from wheat (dataset3).

After peak merging, we got different number of peaks for each replicate of experiment due to experimental variations. Since peak merging results depend on the way to merge, we here provide a table summarizing the number of peaks after peak merging and the difference in two peak merging results (Table 6). Clearly, merging method affects which representative peak to be selected, but not the number of peaks after merging. More detailed explanation of peak merging is provided in Additional file 1.

**Table 6:** The number of peaks: R stands for replicate

**Dataset**	**R1**	**R2**	**R3**	**R4**	**R5**	**R6**	**R7**	**R8**	**R9**	**R10**
Std.	35/78	31/76	28/76	22/75	26/74	23/73	29/74	37/76	37/77	33/75
Mice	55/466	56/456	60/437	51/452	50/418					
Wheat	72/492	62/413	78/493	68/490	79/479	77/521	80/570	59/437		

n1/n2; n2 is the number of compounds after peak merging and this number does not change according to the way of peak merging. However, since each peak merging selects a representative peak by using different criterion, different representative peak sometimes is selected. That is, the way to merge affects representative peak selection for some compounds. n1 is the number of compounds affected by peak merging.

## Competing interests

The authors declare that they have no competing interests.

## Authors’ contributions

JJ, SK and CS conceived and designed this study. JJ and SK developed the program to implement the algorithm. XZ and XS designed and conducted the experiments. All authors read and approved the final manuscript.

## Supplementary Material

Additional file 1 **Supplementary Data I.** This file includes experimental details and detailed explanation of peak alignment methods. Also, more additional performance measures and plots are included. Click here for file

Additional file 2 **Supplementary Data II.** This file includes numerical values for performance measures for all combinations of parameters, i.e, cutoff 1 and cutoff 2. Click here for file

Additional file 3 **Supplementary Data III.** This file includes the number of aligned peaks for all combinations of parameters, i.e, cutoff 1 and cutoff 2. Click here for file

## References

[B1] Wang B Fang A Heim J Bogdanov B Pugh S Libardoni M Zhang X DISCO: distance and spectrum correlation optimization alignment for two-dimensional gas chromatography time-of- flight mass spectrometry-based metabolomics Anal Chem 2010 82 12 5069 5081 10.1021/ac100064b 20476746 PMC2891529

[B2] Ong CY Marriott PJ A review of basic concepts in comprehensive two-dimensional gas chromatography J Chromatogr Sci 2002 40 5 276 291 10.1093/chromsci/40.5.276 12049157

[B3] Shellie R Marriott PJ Comprehensive two-dimensional gas chromatography with fast enantioseparation Anal Chem 2002 74 20 5426 5430 10.1021/ac025803e 12403603

[B4] van den Berg RA Hoefsloot HCJ Westerhuis JA Smilde AK van der Werf MJ Centering, scaling, and transformations: improving the biological information content of metabolomics data BMC Genomics 2006 7 142 10.1186/1471-2164-7-142 PMC1534033 16762068

[B5] Fraga CG Prazen BJ Synovec RE Objective data alignment and chemometric analysis of comprehensive two-dimensional separations with run-to-run peak shifting on both dimensions Anal Chem 2001 73 24 5833 5840 10.1021/ac010656q 11791551

[B6] Mispelaar VG Tas AC Smilde AK Schoenmakers PJ van Asten AC Quantitative analysis of target components by comprehensive two-dimensional gas chromatography J Chematogr A 2003 1019 1–2 15 29 10.1016/j.chroma.2003.08.101 14650601

[B7] Pierce KM Wood LF Wright BW Synovec RE A comprehensive two-dimensional retention time alignment algorithm to enhance chemometric analysis of comprehensive two-dimensional separation data Anal Chem 2005 77 23 7735 7743 10.1021/ac0511142 16316183

[B8] Zhang D Huang X Regnier FE Zhang M Two-dimensional correlation optimized warping algorithm for aligning GCxGC-MS data Anal Chem 2008 80 8 2664 2671 10.1021/ac7024317 18351753

[B9] Kim S Fang A Wang B Jeong J Zhang X An optimal peak alignment for comprehensive two-dimensional gas chromatography mass spectrometry using mixture similarity measure Bioinformatics 2011 27 12 1660 1666 10.1093/bioinformatics/btr188 21493650 PMC3106184

[B10] Jeong J Shi X Zhang X Kim S Shen C Model-based peak alignment of metabolomic profiling from comprehensive two-dimensional gas chromatography mass spectrometry BMC Bioinformatics 2012 13 27 10.1186/1471-2105-13-27 22316124 PMC3323827

[B11] Oh C Huang X Regnier FE Buck C Zhang X Comprehensive two-dimensional gas chromatography/time-of- flight mass spectrometry peak sorting algorithm J Chromatography 2008 1179 2 205 215 10.1016/j.chroma.2007.11.101 PMC3933977 18093607

[B12] Kim S Koo I Fang A Zhang X Smith-Waterman peak alignment for comprehensive two-dimensional gas chromatography mass spectrometry BMC Bioinformatics 2011 12 235 10.1186/1471-2105-12-235 21676240 PMC3133553

[B13] Tusher VG Tibshirani R Chu G Significance analysis of microarrays applied to the ionizing radiation response PNAS 2001 98 9 5116 21 10.1073/pnas.091062498 11309499 PMC33173

[B14] Smith T Waterman M Identification of common molecular subsequences J Mol Biol 1981 147 195 197 10.1016/0022-2836(81)90087-5 7265238

