# Supplementary material for: An efficient post-hoc integration method improving peak alignment of metabolomics data from GCxGC/TOF-MS
Source: BMC Bioinformatics. 2013 Apr 10;14:123. doi: 10.1186/1471-2105-14-123 (PMC3637833; doi:10.1186/1471-2105-14-123)
Supplement: Additional file 1 — Supplementary Data I. This file includes experimental details and detailed explanation of peak alignment methods. Also, more additional performance measures and plots are included. [file 1471-2105-14-123-S1.pdf]

Supplementary Materials: Post-hoc analysis: An efficient integration method improving peak alignment of GCxGC/TOF-MS data with application to various metabolomics data

Supplementary materials consist of as follows. In section 1, we provide experimental details for four datasets such as standard mixture, mice plasma and wheat. Also, detailed description of peak merging is provided in section 1. In section 2, more details about three peak alignment methods are given. In addition, we provide brief comparison among three peak alignment methods such as SW, mSPA and EBM. The definition of four distance measures used in mSPA are given as well. In section 3, additional explanation about algorithm is provided. Then, additional result and corresponding plots are given in section 4. In Sections 5 and 6, manual inspection of alignment results and real life application are given, respectively.

1 Experimental details

1.1 Dataset I: mixture of compound standards

A mixture of 35 amino acids, fatty acids and organic acids were prepared in pyridine. The concentration of each acid in the mixture was 1 mg/mL. A 50 μL aliquot of the mixture was derivatized with 100 μL of N-Methyl-N-(Tert-Butyldimethylsilyl)trifluoroacetamide (MTBSTFA) for 30 min at 60^oC. All GCxGC/TOF-MS analyses were performed on a LECO Pegasus 4D time-of-flight mass spectrometer (TOF-MS) with a Gerstel MPS2 auto-sampler. The Pegasus 4D GCxGC/TOF-MS instrument was equipped with an Agilent 6890 gas chromatograph featuring a LECO two stage cryogenic modulator and secondary oven. A $30m \times 0.25mm\, id. \times 0.25\mu m$ film thickness, Rxi-5ms GC capillary column was used as the primary column for the GCxGC/TOF-MS analysis. A second GC column of $2m \times 0.10mm\ id. \times 0.10\mu m$ film thickness, BPX-50 was placed inside the secondary GC oven after the thermal modulator. The helium carrier gas flow rate was set to 1.0 mL/min at a corrected constant flow via pressure ramps. A $2\mu L$ liquid sample was injected into the liner using the splitless mode with the injection port temperature set at 260^oC. The first-dimension column oven ramp began at 60^oC with a 0.5-min hold after which the temperature was programmed to 280^oC at a rate of $8^oC/min$ and then held at this temperature for 6 min. The second-dimension column temperature was maintained 5^oC higher than the corresponding first-dimension column. The programming rate and hold times were the same for the two columns. The thermal modulator was set to $+20^oC$ relative to the primary oven and a modulation time of 5 s was used. The MS mass range was $45-750m/z$ with an acquisition rate of 200 spectra per second. A 700 s solvent delay was used. The ion source chamber was set at 230^oC with the MS transfer line temperature set to 260^oC and the detector voltage was $1800V$ with an electron energy of $70eV$. The LECO Chro-

maTOF software version 3.41 equipped with the National Institute of Standards and Technology (NIST) MS database (NIST MS Search 2.0, NIST/EPA/NIH Mass Spectral Library; NIST 2002) was used for instrument control, spectrum deconvolution and metabolite identification.

1.2 Dataset II: mice plasma

Metabolites were extracted from a $100\mu L$ mice plasma sample using $900\mu L$ of organic solvent mixture (methanol:water = 8:1). A $50\mu L$ aliquot of plasma extract were further derivatized with N-tert-Butyldimethylsilyl-N-methyltrifluoroacetamide (MTBSTFA). The derivatized metabolite extract was spiked at a concentration of $2.5\mu g/mL$ with a deuterated six component semi-volatiles internal standard (ISTD) mixture prior to GCxGC/TOF-MS analysis by a LECO Pegasus 4D time-of-flight mass spectrometer (TOF-MS). A $30m \times 0.25mm\ id. \times 0.25\mu m$ film thickness, Rxi-5ms, GC capillary column was used as the primary column for the GCxGC/TOF-MS analysis. In the GCxGC configuration, a second column $1.2m \times 0.10mm\ id. \times 0.10\mu m$ film thickness, BPX-50, was placed inside the LECO secondary GC oven after the thermal modulator. Helium carrier gas flow rate was set to $1.0mL$ per minute at a corrected constant flow via pressure ramps. A $1\mu L$ splitless liquid injection was made with the injection port temperature set at 260^oC. The primary column was programmed with an initial temperature of 60^oC for 0.5 minute and then ramped at 7^oC per minute to 315^oC for 8.5 minutes. The secondary column temperature program was set to an initial temperature of 65^oC for 0.5 minute and then ramped at 7^oC per minute to 320^oC with an 8.5 minutes hold time for a total runtime of 45.43 minutes. The thermal modulator was set to $+20^oC$ relative to the primary oven and a modulation time of 5 seconds was used. The MS mass range was $10 - 750m/z$ with an acquisition rate of 150 spectra per second. The ion source chamber was set at 230^oC with the MS transfer line temperature set to 260^oC and the detector voltage was $1800V$ with an electron energy of $-70eV$. The acquired data was processed with a user defined data processing method. The LECO ChromaTOF software version 3.41 equipped with the National Institute of Standards and Technology (NIST) MS database (NIST MS Search 2.0, NIST/EPA/NIH Mass Spectral Library; NIST 2002) was used for instrument control, spectrum deconvolution and metabolite identification.

1.3 Dataset III: wheat

extraction: Around 130mg plant materials (fresh weight from wheat spikelet) was ground with pestle and mortar, then transfer to 2-ml centrifuge tube. One thousand and five hundred μL extraction solution (chloroform : methanol : water = 2 : 4 : 1, precooled at -20^oC), and 60 μL of Ribitol (0.2 mg/mL stock in dH2O) were added , then vortex 10 s. The extraction was carried out on ice for 30 min, and then centrifuge for 10 min at 11,000 g. Transferring the upper phase into a fresh 1.5-ml tube, and drying in a vacuum concentrator without heating.

derivatization: Placing samples stored at -80^oC in a vacuum concentrator for 30 min before derivatization. Add 30 μL 50 mg/ml EH (Ethoxyamine Hydrothoride) solution to the aliquots at 70^oC for 30 min. Then 70 μL MTBSTFA and 10 μL retention index standard mixture (100 μg/mL stock in pyridine) were added to the sample aliquots at 70^oC for 30 min. Add 10 μL 200 μg/mL D6 as internal standards, then the mixture was transferred into glass vials for GCxGC/TOF-MS analysis. What is more, it is essential to prepare one derivatization reaction using an empty reaction tube as a control and centrifugation of the reaction mixture is essential after every incubation step.

GCxGC/TOF-MS parameters: A 2 μL liquid sample was injected at a split ratio of 1:10 into a LECO Pegasus 4D GCxGC/TOF-MS instrument equipped with an Agilent 6890 gas chromatography and a Gerstel MPS2 autosampler, with the injection port temperature set at 280^oC. Chromatography was performed using a non-polar DB-5ms, 60 m x 0.25 mm 1dc x 0.25 μm 1df column, combined with a medium-polar DB-17ms, 1.0 m x 0.1 mm 2dc x 0.1 μm 2df column. The carrier gas was ultra-high purity helium carrier gas (99.999%) with flow rate of 2.0 mL, the ion source chamber was set to 230^oC, the detector voltage was 1700 V, and the electron energy was 70 eV. The thermal modulator was set to +20^oC relative to the primary oven. The second oven was always set to +10^oC with respect to the primary oven. The mass spectra were acquired at a rate of 200 spectra per second with mass range set to m/z = 45-1000. The modulation period for the temperature programmed experiments was set as PM = 2 s, the first dimension column was programmed from 60^oC, 0.5 min ramped to 270^oC, 10 min at 5^oC/min.

raw data reduction: The LECO ChromaTOF software equipped with the National Institute of Standards and Technology (NIST) MS database (NIST MS Search 2.0, NIST/EPA/NIH Mass Spectral Library; NIST 2002), was used for instrument control, spectrum deconvolution, and compound identification. The manufacturer's recommended parameters for ChromaTOF were used to reduce the raw instrument data into a compound peak list. These parameters are: baseline offset = 0.5; smoothing = auto; peak width in first dimension = 30 s; peak width in the second dimension = 0.1 s; signal-to-noise ratio = 50.0; match required to combine peaks = 700; R.T. shift = 0.08 s; minimum forward similarity match = 600. The true peak spectrum was also exported as part of the information for each peak in absolute format of intensity values.

1.4 Dataset IV: mice diet data

Mice were exposed to tap water for one week prior to initiating feeding with either low fat diet (13% fat in calories) or high fat diet (42% fat in calories) (Harlan Laboratories, Madison, WI) for 10 weeks. Two different treatment groups were evaluated in this study: 6 mice fed a low fat diet and tap water (sample group LFD+tap); 5 mice fed a high fat diet and tap water (sample group HFD+tap).Food and water consumption were measured twice a week. Body weight was measured once a week. For termination, mice were anesthetized with ketamine/xylazine (100/15 mg/kg i.m.). Portions of liver tissue were frozen

immediately in liquid nitrogen.

A sample of liver tissue was weighed and then homogenized for 2 min after adding water at a ratio of 100 mg liver tissue/mL water. The homogenized sample was then stored at -80^oC until use. A 100 μL of liver sample and 400 μL methanol were mixed and vortexed for 1 min followed by centrifugation at room temperature for 10 min at 15000 rpm. 400 L of the supernatant was aspirated into a plastic tube and dried by N2 flow. The metabolites extracts were then dissolved in 40 μL ethoxyamine hydrochloride solution (30 mg/mL) and vigorously vortex-mixed for 1 min. Methoxymation was carried out at 70^oC for 1 hour. After adding 40 L N-(tert-butyldimethylsilyl)-N-methyltrifluoroacetamide (MTBSTFA) mixed with 1% tert-Butyldimethylchlorosilane (TBDMSCI), derivatization was carried out at 70^oC for 1 hour. Stock solutions were then transferred to GC vials for analysis. The methoxymation and derivatization were prepared just before GCxGC/TOF-MS analysis. The LECO Pegasus 4D GCxGC/TOF-MS instrument was equipped with an Agilent 6890 gas chromatograph and a Gerstel MPS2 auto-sampler (GERSTEL Inc., Linthicum, MD), featuring a LECO two-stage cryogenic modulator and secondary oven. The primary column was a 60 mx0.25 mm 1dc x 0.25 μm 1df, DB-5ms GC capillary column (phenyl arylene polymer virtually equivalent to a (5%-phenyl)-methylpolysiloxane). A second GC column of 1 m x 0.25 mm 1dc x 0.25 μm 2df, DB17ms ((50%-phenyl)-methylpolysiloxane) was placed inside the secondary GC oven after the thermal modulator. Both columns were obtained from Agilent Technologies (Agilent Technologies J&W, Santa Clara, CA). The helium carrier gas (99.999% purity) flow rate was set to 1.0 mL/min at a corrected constant flow via pressure ramps. The inlet temperature was set at 280^oC. The primary column temperature was programmed with an initial temperature of 60^oC for 0.5 min and then ramped at 5^oC/min to 280^oC and kept for 12 min. The secondary column temperature program was set to an initial temperature of 70 ^oC for 0.5 min and then also ramped at the same temperature gradient employed in the first column to 280 ^oC accordingly. The thermal modulator was set to +20 ^oC relative to the primary oven, and a modulation time of PM = 2.5 s was used. The mass range was set as 45-1000 m/z with an acquisition rate of 200 mass spectra per second. The ion source chamber was set at 230^oC with the transfer line temperature set to 280^oC, and the detector voltage was 1680 V with electron energy of 70 eV. The acceleration voltage was turned on after a solvent delay of 775 s. The split ratio was set at 40:1.

1.5 Preprocessing

Prior to metabolite identification and peak alignment, we did another preprocessing including peak merging. First of all, we removed compounds without name by ChromaTOF, i.e., labeled "Unknown". After that, in case of multiple peaks, we merged peaks in terms of peak area or peak similarity. The whole process of handling data is represented in Figure 1.

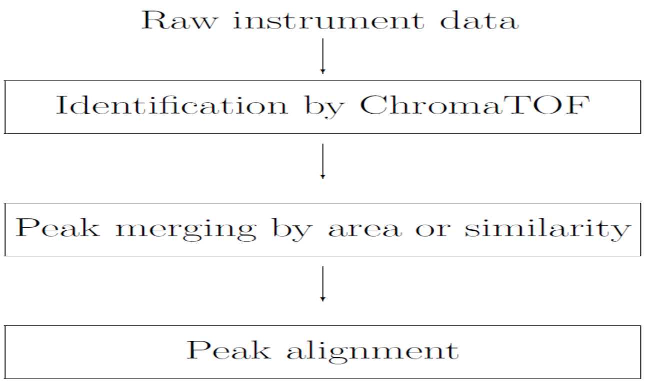

Figure 1: Graphical representation of work flow of GCxGC/TOF-MS data prior to post-hoc analysis.

1.5.1 Peak merging

Theoretically, a peak is generated by a compound. However, multiple peaks occur practically. To remedy such problem, we consider peak merging. There are two common ways of peak merging: peak merging by peak area and peak merging by peak similarity. Since we select the peak with biggest number in terms of peak area or similarity, both merging may result in different results. As an illustrating example, we look at a experimental output from a mixture of standard compounds and focus on the compound with multiple peaks called Pyridine (CAS: 110-86-1). Five peaks for the compound are summarized in the Table 1. As a representative peak, we select the first one if we use area-based peak merging. However, we get the second one if we use similarity-based peak merging.

Table 1: Peak merging

Name	CAS	RT1	RT2	Area	Similarity
Pyridine	110-86-1	369.719	1.162	**28831918**	943
Pyridine	110-86-1	379.711	1.175	2788666	**948**
Pyridine	110-86-1	384.707	1.188	925142	931
Pyridine	110-86-1	389.704	1.208	548115	914
Pyridine	110-86-1	394.7	1.214	569849	882

Standard mixture data; peak merging by Area or Similarity

Even though the representative compound is different according to the way of peak merging, the number of aligned peaks are the same and are summarized in Table 2. Also, the difference in merging results are summarized in Table 3

Table 2: Number of peaks after/before peak merging

Run ID	${R_1}^1$	${R_2}^1$	${R_3}^1$	${R_4}^1$	${R_5}^1$
N	78/183	76/188	76/163	75/152	74/154
Run ID	${R_6}^1$	${R_7}^1$	${R_8}^1$	${R_9}^1$	${R_{10}}^1$
N	73/147	74/175	76/164	77/171	75/175
Run ID	${D_1}^2$	${D_2}^2$	${D_3}^2$	${D_4}^2$	${D_5}^2$
N	466/759	456/733	437/695	452/727	418/661
Run ID	${D_1}^3$	${D_2}^3$	${D_3}^3$	${D_4}^3$	${D_5}^3$
N	492/798	413/637	493/831	490/795	479/802
Run ID	${D_6}^3$	${D_7}^3$	${D_8}^3$		
N	521/855	570/979	437/717		

Superscript represents dataset: 1,2,3 presents std. mixture, mice and wheat, respectively; subscript represents replicates

Table 3: Difference between both peak merging ways

Dataset	R1	R2	R3	R4	R5	R6	R7	R8	R9	R10
Std.	35/78	31/76	28/76	22/75	26/74	23/73	29/74	37/76	37/77	33/75
Mice	55/466	56/456	60/437	51/452	50/418					
Wheat	72/492	62/413	78/493	68/490	79/479	77/521	80/570	59/437		

n1/n2; n2 is the number of compounds after peak merging and n1 is the number of different compounds selected by both peak merging for each replication.

2 Methods and distance measures

2.1 Three existing methods

2.1.1 Smith-Waterman (SW)

Smith and Waterman (1981) developed a general method for identification of molecular subsequences. Kim et al. (2011) modified the traceback process of the SW method and proposed three variants of the algorithm: SW repeat alignment with maximum scores (SWRM), SW repeat alignment with ending scores (SWRE) and SW repeat alignment with maximum of ending scores (SWRME). Then, they applied the method to GCxGC/TOF-MS data for the purpose of metabolite alignment.

Suppose that we have two sequences $X = x_1, \cdots, x_m$ and $Y = y_1, \cdots, y_n$ to compare. We denote the subsequence of X and Y by $X_{h,i} = x_h, \cdots, x_i$ and $Y_{k,j} = y_k, \cdots, y_j$, respectively where $1 \le h \le i \le m$ and $1 \le k \le j \le n$. The SW algorithm produces a matrix H representing the degree of similarity with a

boundary condition. Each element of the matrix H consists of

$$H(i,j) = \max\{H_{i-1,j-1} + w_{i,j}, H_{i-1,j} - d, H_{i,j-1} - d, 0\}$$

where $w_{i,j}$ is similarity function and d is gap penalty and $H(i,0) = 0 = H(0,j)$. Note that all elements of H are nonnegative.

In the context of peak alignment, X and Y are peak lists to align and the similarity function w is defined:

$$w_{i,j} = u \cdot 1_{S(x_i,y_j)\geq\rho_c} + v \cdot 1_{S(x_i,y_j)<\rho_c}$$

where u and v are non-negative constants, $S(x_i, y_j)$ is the spectral similarity between two peaks x_i and y_j, and ρ_c is cutoff value of spectral similarity. As a spectral similarity measure, Pearson's correlation coefficient was employed.

2.1.2 mSPA

The method consists of two main algorithms: peak matching and parameter optimization. As a similarity measure for peak matching, they defined a mixture similarity score (M_d). Given target (T) and reference (R) peak lists, the mixture score between target peak t_j and reference peak r_i is defined:

$$M_d(t_j, r_i) = \frac{w}{1 + D_d(t_j, r_i)} + (1 - w)S(t_j, r_i)$$

where $w(0 \leq w \leq 1)$ is weight, S is spectral similarity measure and $D_d(d = 1, 2, 3, 4)$ presents distance measure. They considered four different distance measures: Euclidean (D_1), Maximum (D_2), Manhattan (D_3) and Canberra (D_4). Definitions of those distance measures are provided in Additional file 1. While they consider four different distance measures of retention time, they consider only two spectral similarity measures such as dot product and Pearson correlation. The reason is that Liu et al. (2007) compared several spectral similarity measures and concluded that Pearson's correlation coefficient and dot product performed relatively well. Thus, they used dot product as a default spectral similarity measure and added Pearson's correlation coefficient as an option.

Peak matching: Given an experiment pair, peak matching is performed based on similarity score. To find the best matching reference peak for target peak t_j, mixture scores between each of all reference peaks (R) and target peak t_j are calculated and the best matching reference peak is selected by

$$r_i = argmax_{r_h \in R} M_d(t_j, r_h|w)$$

where R is reference peak list and d presents distance measure. They repeat the same peak matching for all target peaks.

Parameter optimization: There are two parameters in the formula of mixture score: $\theta = (w, d)$. For parameter optimization, they defined an ad-hoc

likelihood-type function:

$$f(T, R|\theta) \equiv \sum_{k=1}^{l} \left[M_d(t_k, r_k|w) + S(t_k, r_k) + \frac{1}{1 + D_d(t_k, r_k)} \right]$$

where T and R are target and reference peak lists, respectively and l is the number of all matched peaks between T and R. The optimal estimates of weight and distance measure are obtained by

$$\hat{\theta} = argmax_{\theta} f(T, R|\theta).$$

2.1.3 Empirical Bayes model

Jeong et al (2011) developed a hierarchical statistical model (EBM) for metabolite identification and peak alignment in an unified framework for comprehensive two-dimensional GC mass spectrometry data. To address the nature of the database search algorithm, the model consists of four layers: (1) marginal probability that each compound in library (reference) exist in sample (target) is calculated (2) depending on the existence/absence information of the compound, different conditional probability of the compound being matched to a compound in sample are calculated. (3) based on the information from previous layers, the conditional probability that the match is correct is calculated. (4) based on the decision, we separate the scores and estimate two score densities: true positive and true negative score densities. More detailed description of the model was provided in Jeong et al (2011). Here, we introduce the model briefly.
Layer 1: The marginal probability that each metabolite in the reference is present in target is represented:

$$\mathrm{P}(Y_j = 1) = \rho, \quad j = 1, 2, \cdots, N, \tag{1}$$

where N is the number of the peaks in the reference.
Layer 2: According to the value of Y_j, we consider two different conditional probabilities: $\mathrm{P}[Z_j = 1|Y_j = 0]$ and $\mathrm{P}[Z_j = 1|Y_j = 1]$. Note that even though a metabolite j does not exist in target ($Y_j = 0$), there is some chance for the metabolite to be claimed as present ($\mathrm{P}[Z_j = 1|Y_j = 0] > 0$). The following model for the case $Y_j = 0$ is considered:

$$\mathrm{P}[Z_j = 1|Y_j = 0] = {\eta_0}^{I(b_j=0)} \gamma(\beta; b_j)^{I(b_j>0)}, \tag{2}$$

where $\gamma(\beta; b_j) = 1 - \frac{1}{1+\exp{(\beta_0+\beta_1 b_j+\beta_2 {b_j}^2)}}$. The b_j is defined using the metabolite reference in the following way:

$$b_j = \sum_{k \neq j, k \in C, I(r_{kj} < h)} 1/a_k, \tag{3}$$

where $a_k = \sum_{q \in C} I(r_{qk} < h)$, r_{qk} is a mixture similarity score between peaks q and k in the reference, C is the set of peaks in the reference, and $I(\cdot)$ is the indicator function.

Similarly, for the case $Y_j = 1$, we consider the following model:

$$P[Z_j = 1|Y_j = 1] = {\eta_1}^{I({b_j}^*=1)} \lambda(\alpha; {b_j}^*)^{I({b_j}^*>1)}, \tag{4}$$

where $\lambda(\alpha; {b_j}^*) = 1 - \frac{1}{1+\exp(\alpha_0+\alpha_1 {b_j}^* + \alpha_2 {b_j}^{*2})}$. The ${b_j}^*$ is defined:

$${b_j}^* = \sum_{k \in C, I(r_{kj}<h)} 1/a_k \tag{5}$$

where ${b_j}^*$ includes metabolite j itself as a neighbor to account for the fact that $Y_j = 1$.

Layer 3: For reference metabolites matched to at least one target metabolite, we calculate the correctness of those matches, i.e., conditional probability of W_{jl} given Y_j and Z_j. More specifically, the probability for those matches of metabolite j with $Y_j = 1$ and $Z_j = 1$ to be correct is given:

$$P(W_{jl} = 1|Y_j = 1, Z_j = 1) = \tau. \tag{6}$$

Note that our matching is not always correct even though metabolite j is true positive.

Layer 4: To characterize the distribution of the similarity scores, we consider parametric approach, i.e., normal mixture:

$$f(S_j|W_j) = \prod_l f_T(S_{jl}; \phi_T)^{W_{jl}} f_F(S_{jl}; \phi_F)^{(1-W_{jl})}, \tag{7}$$

where f_T and f_F are the distributions of the scores of the correct matches and incorrect matches, respectively, f is the mixture of them and ϕ_T and ϕ_F are corresponding parameters such as mean and variance.

Matching confidence for peak alignment: When matching peaks, Jeong et al. (2012) used the posterior probability that the match is correct. That is, the matching confidence of metabolite j in reference to a target metabolite can be calculated as the posterior probability of W_{jl}:

$$P_{jl} = P[W_{jl} = 1|Z_j = 1, S_j; \hat{\theta}] \tag{8}$$

where $\hat{\theta}$ is the estimated parameter vector.

2.2 Brief comparison

All methods mentioned in main text use peak lists obtained from instrument control software ChromaTOF as input data. However, they are different in several respects. First of all, while SW and EBM are able to process both homogeneous and heterogeneous data, mSPA can be applied to homogeneous data only. Second, as a spectral similarity measure, Pearson's correlation coefficient, dot product and cosine angle are used in SW, mSPA and EBM respectively. Third, as a retention time similarity measure, Euclidean norm and elution order difference are used in SW and EBM, respectively. On the other hand,

mSPA considers 4 different distance measures: Euclidean(D_1), Maximum(D_2), Manhattan(D_3) and Canberra(D_4). Finally, even though they use both spectral similarity and RT distance as a measure of peak similarity, the way both information are used is different. mSPA and EBM use a weighted average of RT distance and spectral similarity. The Table 4 summarizes the differences among methods.

Table 4: Summary of difference among methods

Method	Data	Spectral measure	RT measure	Peak matching
SW	both	Pearson's corr.	Euclidean norm	Spectra similarity
mSPA	homo	dot product	canverra	weighted average
EBM	both	cosine angle	rank difference	weighted average

For mSPA, dot product and canverra are default value of spectral similarity and retention time distance, respectively

More comparison results are provided in Kim et al. (2011) and Jeong et al. (2011).

2.3 Distance measures

The definitions of the four different distance measures, which were used in Kim et al., are given: Euclidean distance (D_1), Maximum (aka Chebyshev) distance (D_2), Manhattan distance (D_3), and Canberra distance (D_4).

$$D_1(y_j, x_i) = \sqrt{(y_{(j,1)} - x_{(i,1)})^2 + (y_{(j,2)} - x_{(i,2)})^2} \tag{9}$$

$$D_2(y_j, x_i) = \max\left(|y_{(j,1)} - x_{(i,1)}|, |y_{(j,2)} - x_{(i,2)}|\right) \tag{10}$$

$$D_3(y_j, x_i) = |y_{(j,1)} - x_{(i,1)}| + |y_{(j,2)} - x_{(i,2)}| \tag{11}$$

$$D_4(y_j, x_i) = \frac{|y_{(j,1)} - x_{(i,1)}|}{|y_{(j,1)} + x_{(i,1)}|} + \frac{|y_{(j,2)} - x_{(i,2)}|}{|y_{(j,2)} + x_{(i,2)}|} \tag{12}$$

where $y_{(j,1)}$ and $x_{(i,1)}$ are the first dimension retention time of the peaks y_j and x_i, and $y_{(j,2)}$ and $x_{(i,2)}$ are the second dimension retention time of the peaks y_j and x_i.

3 Algorithm

Here is additional information for pairwise post-hoc algorithm. Let's denote set of peak pairs aligned by Naive method by AP_N and that of peak alignment method by AP_M. Then, peak pairs in common area (CA) are represented by CA= $AP_N \cap AP_M$ and two disjoint ares (DA1 and DA2) are represented by DA1= $AP_N - AP_M$ and DA2= $AP_M - AP_N$, respectively. At this stage, we have potential true positive alignment set (TPAS) including all pairs in CA.

Suppose that there are k_1 aligned peaks in DA1, $\{p_{11}, \cdots, p_{1k_1}\}$ and k_2 in DA2, $\{p_{21}, \cdots, p_{2k_2}\}$. Given similarity scores for those pairs, we apply cutoff1 value to all peak pairs in DA1 and DA2. Then, some pairs surviving cutoff are added to TPAS.

Graphical representation of how to apply two important values (cutoff1 and cutoff2) is given in Figure 2.

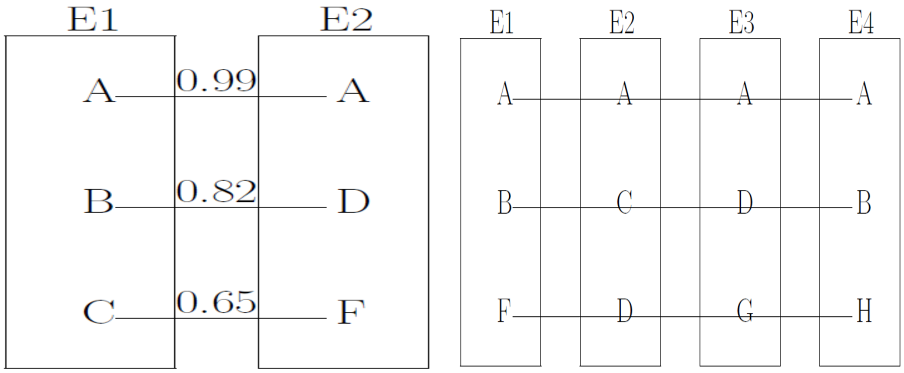

Figure 2: Graphical representation of cutoff1 and cutoff2. Cutoff1 value is applied to a pair of experiment (left panel). For example, if cutoff1=0.95 is used, then the first peak pair with the score of 0.99 is kept and the other two discarded. Cuoff2 value is applied to global alignment only. For example, given cutoff2=3, one of three aligned compounds is considered as correctly aligned (right panel).

Pairwise alignment is given in the left panel of the Figure 2. The numbers in the figure is similarity score. The aligned peaks with score bigger than cutoff1 value (say 0.95) are kept and others are removed. For example, only one peak with score of 0.99 (i.e., A-A peak pair) is aligned in the figure. For global alignment, let's assume we got global alignment results in right panel of the same figure. If we use cutoff2=3, then the aligned peak with the same name for more than 3 compounds is kept and others are not. In this example, the only one (i.e., A-A-A-A) is considered as globally aligned.

4 Results

We considered two different types of peak merging: peak merging by peak area and similarity. Also, we considered two different performance measures: distance-based measure (Euclidean distance) and variation-based measure (CV). In other words, we considered four different scenarios. Since all results plots corresponding area-based peak merging are given in main article, we here provide two tables summarizing post-hoc results (see Tables 6 and 7). For other three

cases, all corresponding tables summarizing post-hoc results are given in Additional file 2.

We also calculated the number of aligned peaks by each method. For pairwise comparison, we calculated median value of the number of peak pairs for all possible pairs. For global comparison, the cutoff2 where all aligned compounds have the same name was selected. Here we provide a table summarizing the number of peak pairs when cutoff1 = 0.99 (Table 5). Other results are summarized in Additional file 3.

Table 5: The number of peak pairs after post-hoc analysis when cutoff1=0.99; peak merging by area/peak merging by similarity

	pairwise			global		
	EBM	mSPA	SW	EBM	mSPA	SW
Std	61/62	71/70	69/69	44/41	63/63	53/46
Mice	142/142	166/172	157/153	65/66	87/89	33/30
Wheat	170/179	232/234	192/191	68/76	84/87	30/27

4.1 Peak merging by peak area

4.1.1 Distance-based performance measure

Since results plots for distance-based measure are provided in main article, here we provide two tables (Table 6 for distance-based measure and Table 7 for variation-based measure when cutoff1=0.99) summarizing performance measures.

In case of mice data, pairwise trace plot using distance-based performance measures (RT only) is given in Figure 3

On the other hand, corresponding box plot for global alignment is given in Figure 4

In addition, variation-based performance measures are provided in Additional files 2 and 3. Also, all corresponding measures for similarity-based peak merging are provided in Additional files 2 and 3. However, all corresponding plots are available on request.

5 Manual validation of alignment results

In this section, we do manual inspection to check if posthoc method improves alignment. For simplicity, we focus on a pair of experiment from standard mixture data. Given the experiment pair, we apply our pairwise posthoc algorithm to the data and compare alignment results before and after posthoc. There are 78 and 76 peaks in each dataset, respectively. EBM, which is a model-based alignment algorithm, produces 67 aligned peaks and then we got 59 aligned

Table 6: Pairwise: average of distance-based measures over all pairs: before/after post-hoc analysis when cutoff1=0.99

		Std. mixture		
Method	RT1	RT2	MRT	RT
EBM	16.3396/2.4841	0.0202/0.0148	8.1799/1.2494	16.3451/2.4902
mSPA	5.9595/6.0595	0.0172/0.0185	2.9884/3.0390	5.9654/6.0653
SW	1.4544/1.7445	0.0115/0.0099	0.7329/0.8772	1.4624/1.7505
Naive	7.3554	0.0243	3.6898	7.3613
		Rat		
Method	RT1	RT2	MRT	RT
EBM	81.7213/13.4712	0.0615/0.0264	40.8914/6.7488	81.7301/13.4839
mSPA	15.7982/5.5890	0.0304/0.0211	7.9143/2.8050	15.8091/5.6013
SW	0.7948/6.8691	0.0620/0.0190	0.4284/3.4440	0.8483/6.8820
Naive	73.4044	0.0679	36.7361	73.4156
		Wheat		
Method	RT1	RT2	MRT	RT
EBM	158.1612/25.4131	0.0474/0.0124	79.1043/12.7127	158.1628/25.4150
mSPA	25.9809/6.6417	0.0164/0.0059	12.9986/3.3238	25.9831/6.6434
SW	1.9515/6.5940	0.0314/0.0072	0.9915/3.3006	1.9651/6.5964
Naive	160.0528	0.0424	80.0476	160.0547

peaks after posthoc with cutoff1 value of 0.99, i.e., there are 8 peak pairs removed after posthoc. The list of 8 removed peak pairs are given in Table 8.

After manual inspection, we noticed that 6 of 8 aligned pairs, which are removed by post-hoc, are properly removed because their alignment are possibly wrong. That is, our post-hoc approach works well. Here we select 2 of them (i.e., one (4th row) is correct decision of our post-hoc and the other (2nd row) incorrect), and provide corresponding raw chromatogram 3D plot (Figure 5).

6 Application to biomarker discovery

We applied global post-hoc to mouse diet data with two groups: HFD(5) and LFD(6). Global alignment results are summarized in Table 9.

With cutoff1=0.99 and Cutoff2=11, we got 44 aligned metabolites. We then applied SAM to the 44 metabolites and found 15 statistically significant metabolites. The list of 15 biomarker metabolites, which are found at the FDR level of 0.05, is given in Table 4 in main text. Here we provide two SAM plots at FDR=0.05 and 0.20 (Figure 6). Metabolites (abundance of HFD is higher) are denoted by dot in red and metabolites (abundance of LFD is higher) are denoted by dot in green.

Table 7: Pairwise: average of variation-based measures over all pairs: before/after post-hoc analysis when cutoff1=0.99

	Std. mixture			
Method	RT1	RT2	MRT	RT
EBM	0.0086/0.0019	0.0079/0.0054	0.0082/0.0037	0.0082/0.0037
mSPA	0.0029/0.0029	0.0067/0.0071	0.0048/0.0050	0.0048/0.0050
SW	0.0013/0.0016	0.0043/0.0038	0.0028/0.0027	0.0028/0.0027
Naive	0.0038	0.0093	0.0066	0.0066
	Rat			
Method	RT1	RT2	MRT	RT
EBM	0.0395/0.0065	0.0320/0.0131	0.0358/0.0098	0.0358/0.0098
mSPA	0.0086/0.0028	0.0157/0.0104	0.0122/0.0066	0.0122/0.0066
SW	0.0004/0.0033	0.0334/0.0093	0.0169/0.0063	0.0169/0.0063
Naive	0.0367	0.0353	0.0360	0.0360
	Wheat			
Method	RT1	RT2	MRT	RT
EBM	0.0641/0.0103	0.0339/0.0084	0.0490/0.0094	0.0490/0.0094
mSPA	0.0117/0.0033	0.0125/0.0043	0.0121/0.0038	0.0121/0.0038
SW	0.0011/0.0029	0.0235/0.0051	0.0123/0.0040	0.0123/0.0040
Naive	0.0651	0.0309	0.0480	0.0480

Table 8: 8 Aligned peak list removed after posthoc when cutoff1=0.99

Exp1	Exp2	Correct/incorrect
Benzene, 1,3-dichloro-	Benzene, 1,2-dichloro-	correct
Benzene, 1,2-dichloro-	Benzene, 1,3-dichloro-	incorrect
Tridecane	Dodecane	correct
Pentadecane	Tetradecane	correct
Octadecane	Heptadecane	correct
Anthracene	Phenanthrene	correct
Nonadecane	Octadecane	incorrect
Eicosane	Nonadecane	correct

In case of global alignment of diet data, box plots using distance-based performance measures (EBM only) are given in Figure 7 and corresponding box plots for cv-based performance measures are given in Figure 8

For comparison, results before/after post-hoc are summarized in Tables 10 and 11, respectively.

The three metabolites without * are removed by post-hoc. The reason is that even though they are aligned by EBM, assigned names are different. For

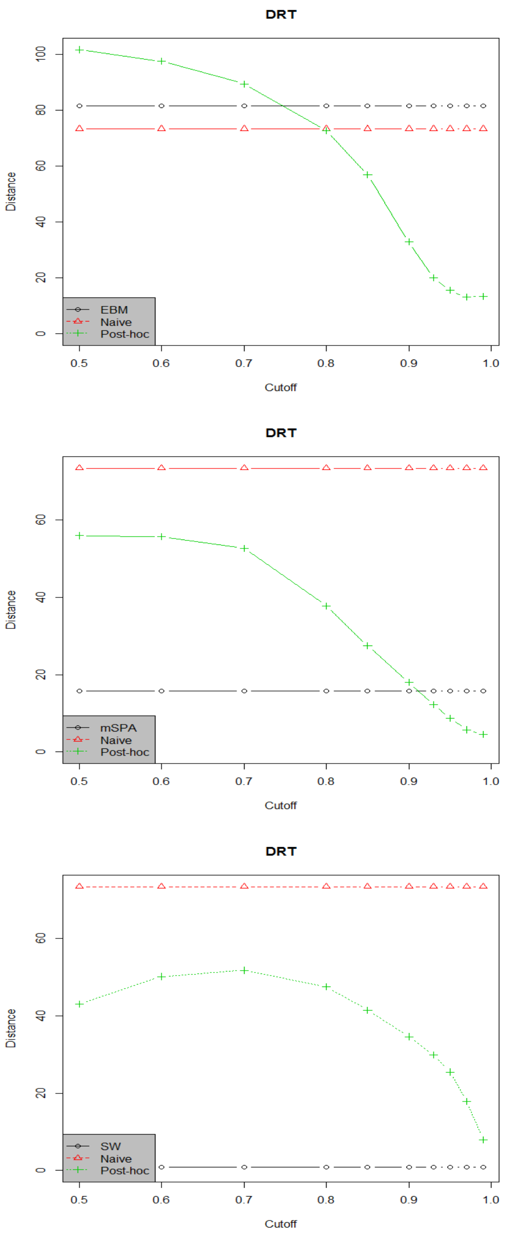

Figure 3: Mice data (pairwise): trace plot of EBM (top), mSPA (center) and SW (bottom).

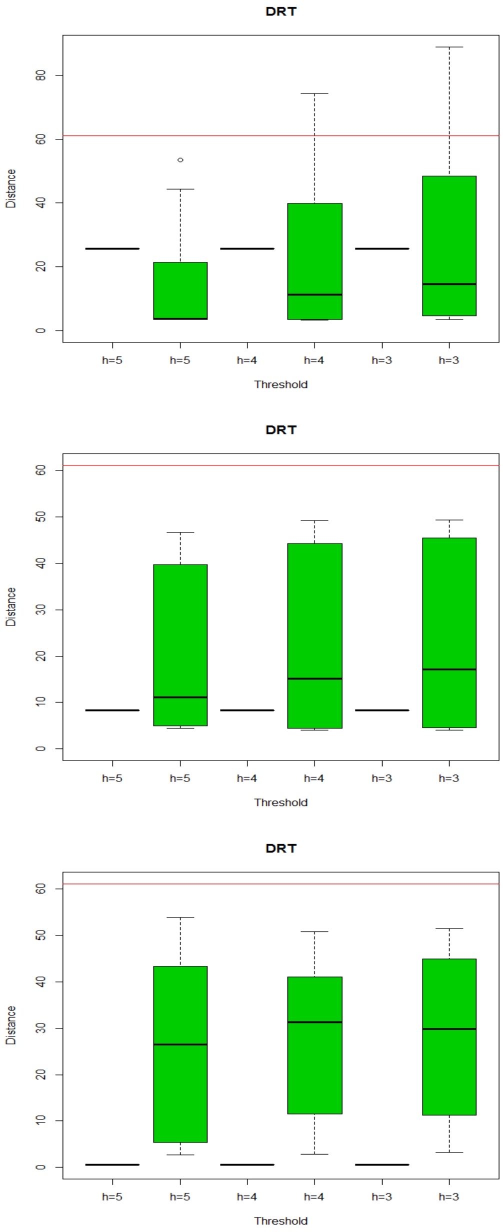

Figure 4: Mice data (global): trace plot of EBM (top), mSPA (center) and SW (bottom).

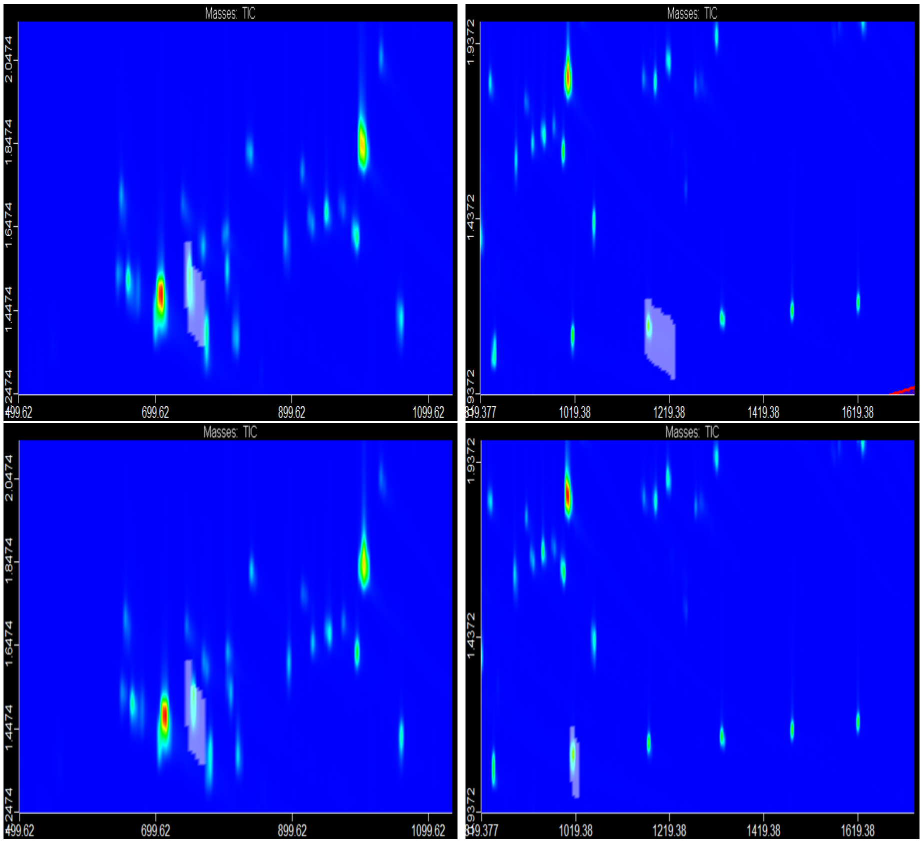

Figure 5: Chromatogram 3D plot for manual inspection: two plots in left column correspond to the second row in Table 8. This alignment is possibly correct even though our post-hoc removed them. Two plots in right column correspond to the fourth row in Table 8. This alignment is possible wrong and our post-hoc correctly removed them.

example, the first aligned peak consists of 5 Ethanol (CAS:2916-68-9), 4 Silanol (CAS:1066-40-6), 1 Propane (CAS:102-52-3) and 1 tert-Butyldimethylsilanol (CAS:18173-64-3). The second one from the bottom consists of 7 L-Lysine (CAS: 107715-99-1) and 4 2-Piperidinecarboxylic acid (CAS:114454-65-8). The last one from the bottom consist of 10 Pentasiloxane (CAS:141-63-9) and 1 Trisiloxane (CAS:3555-47-3).

Table 9: Diet data (series): number of aligned peaks when cutoff1=0.95 and 0.99 for each threshold.

	Cutoff1=0.95	Cutoff1= 0.99
Cutoff2=11	44	44
Cutoff2=10	49	44
Cutoff2=9	50	44
Cutoff2=8	53	44
Cutoff2=7	54	44
Cutoff2=6	54	44

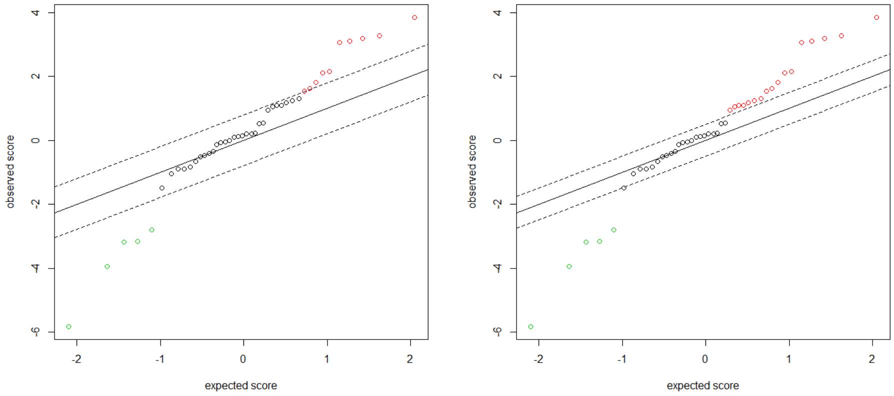

Figure 6: Diet data (global): SAM plot at FDR=0.05 (left) and FDR=0.25 (right).

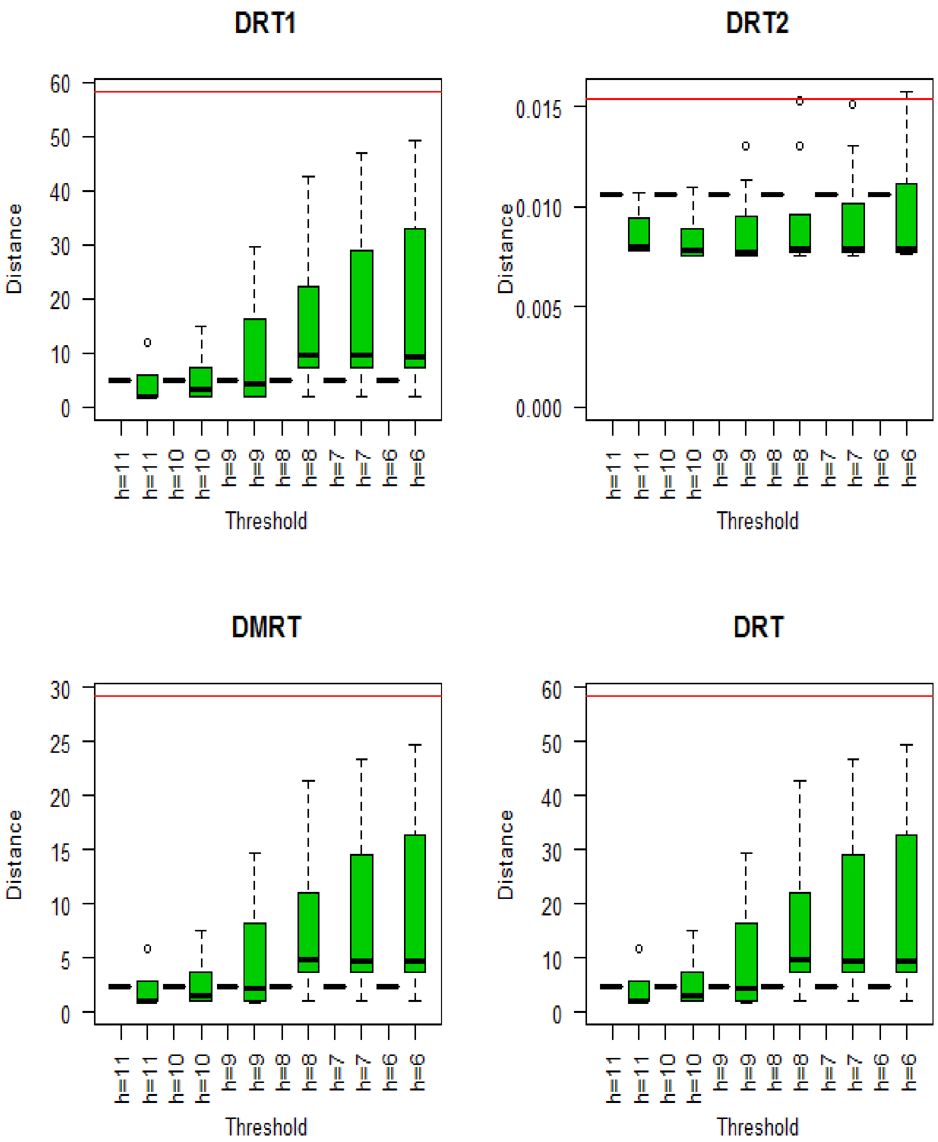

Figure 7: Diet data (global): distance-based performance measure of EBM. The solid line in red presents Naive method. Each threshold value corresponds to two box plots, i.e., before/after post-hoc and each box plot after post-hoc is made by using 10 numerical values corresponding to each cutoff1 values.

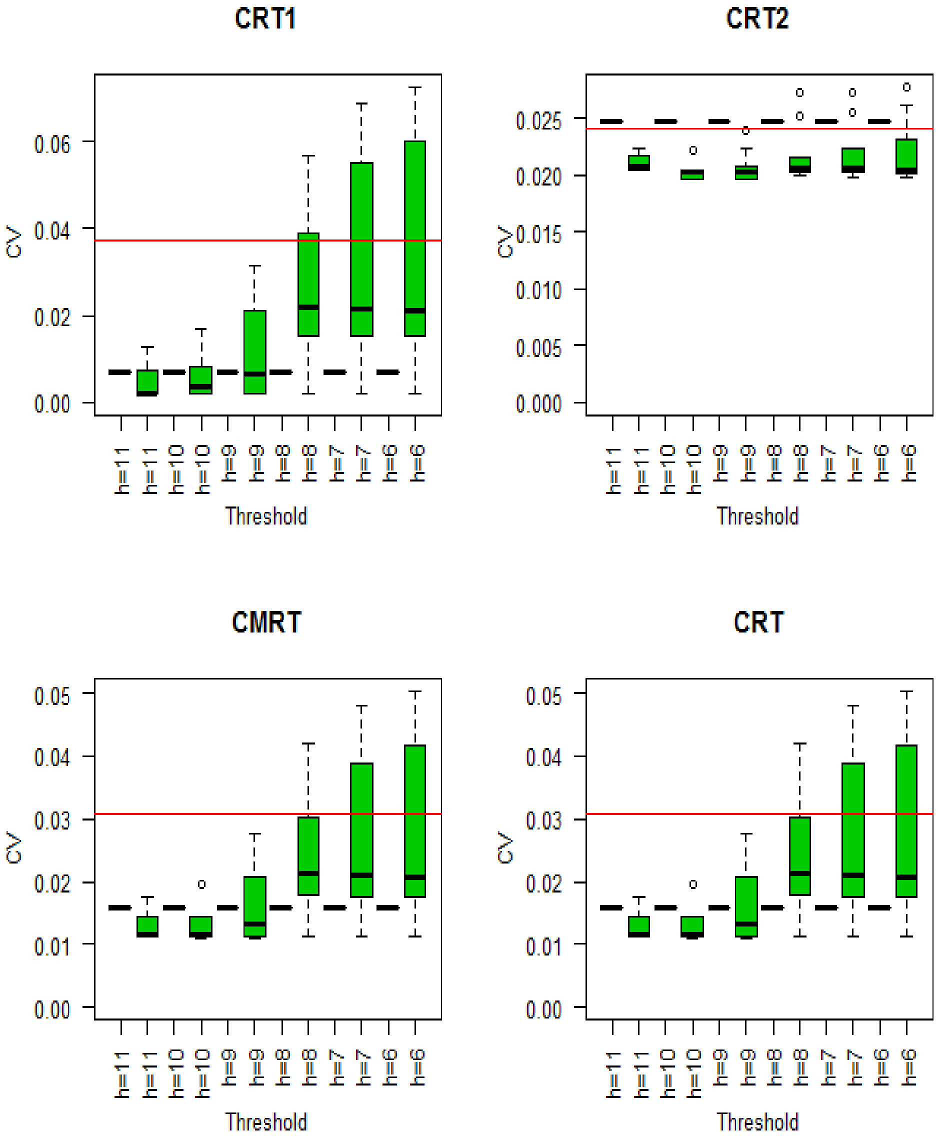

Figure 8: Diet data (global): cv-based performance measure of EBM. The solid line in red presents Naive method. Each threshold value corresponds to two box plots, i.e., before/after post-hoc and each box plot after post-hoc is made by using 10 numerical values corresponding to each cutoff1 values.

Table 10: Diet data (series): peak alignment method EBM is applied to diet data with two group (HFD and LFD) and then SAM is applied to 49 globally aligned metabolites. By SAM, 13 biomarker metabolites are found. For 7 of them, the abundance of HFD is significantly higher than that of LFD (top 7 metabolites).

Name	CAS	SAM score
Ethanol	2916-68-9	4.1732
Acetophenone	98-86-2	3.4157*
Palmitelaidic acid	82326-15-6	3.2679*
N,N-Diethyl-1,1,1-trimethylsilylamine	996-50-9	3.1105*
Tetradecanoic acid	104255-79-0	2.3302*
L-Phenylalanine	107715-95-7	2.1520*
Arachidonic acid	113516-18-0	1.8544*
Pyridine	110-86-1	-5.8398*
Cyclotrisiloxane, hexamethyl-	541-05-9	-3.9723*
Dodecanoic acid	104255-77-8	-3.3729*
Ethanamine	16654-64-1	-3.1949*
L-Lysine	107715-99-1	-3.0462
Pentasiloxane	141-63-9	-2.1794

Table 11: Diet data (series): Global post-hoc with cutoff1=0.99 and cutoff2=11 is applied to diet data with two group (HFD and LFD) and then SAM is applied to 44 globally aligned metabolites. By SAM, 15 biomarker metabolites are found. For 10 of them, the abundance of HFD is significantly higher than that of LFD (top 10 metabolites). Compared to the results before post-hoc, we got more biomarker metabolites. 10 common biomarker metabolites in both results are denoted by * in the last column.

Name	CAS	SAM score
Ethanol	2916-68-9	4.1732
Acetophenone	98-86-2	3.4157*
Palmitelaidic acid	82326-15-6	3.2679*
N,N-Diethyl-1,1,1-trimethylsilylamine	996-50-9	3.1105*
Tetradecanoic acid	104255-79-0	2.3302*
L-Phenylalanine	107715-95-7	2.1520*
Arachidonic acid	113516-18-0	1.8544*
Pyridine	110-86-1	-5.8398*
Cyclotrisiloxane, hexamethyl-	541-05-9	-3.9723*
Dodecanoic acid	104255-77-8	-3.3729*
Ethanamine	16654-64-1	-3.1949*
L-Lysine	107715-99-1	-3.0462
Pentasiloxane	141-63-9	-2.1794
